# Effectiveness of suicide means restriction: an overview of systematic reviews

**DOI:** 10.1136/bmjment-2025-302069

**Published:** 2025-12-09

**Authors:** Sarah Steeg, Sarah Ledden, Lisa Marzano, Rina Dutta, Leah Quinlivan, Nav Kapur, Ann John, Roger Thomas Webb

**Affiliations:** 1 Division of Psychology and Mental Health, The University of Manchester, Manchester, England, UK; 2 Population Health Improvement UK (PHI-UK), London, UK; 3 King’s College London, London, England, UK; 4 Psychology, Middlesex University London - London Campus, London, England, UK; 5 NIHR Greater Manchester Patient Safety Research Collaboration, University of Manchester, Manchester, UK; 6 Swansea University, Swansea, West Glamorgan, UK

**Keywords:** Psychosocial Intervention, Mental Health

## Abstract

**Question:**

Means restriction for suicide prevention at a population level typically involves policy or environmental changes to limit access to suicide methods. Several systematic reviews of suicide means restriction exist. This umbrella review aimed to synthesise their findings, assess evidence quality, quantify primary study overlap and identify evidence gaps.

**Study selection and analysis:**

Searches were conducted across Web of Science, Ovid (PsycINFO, EMBASE), Cochrane and PubMed, supplemented by reference list screening. Study quality was assessed using A MeaSurement Tool to Assess systematic Reviews-2. Study overlap was calculated using the corrected covered area.

**Findings:**

We included 20 systematic reviews, synthesising evidence from 179 unique primary studies. Physical barriers to prevent jumping showed strong effect sizes, although primary study overlap was high. Train platform screen doors were associated with reduced site-specific suicide mortality, with no evidence of displacement to other sites, although the number of studies was small. Paracetamol pack size limitation reduced self-poisoning admissions, with mixed impacts on mortality. Bans on highly hazardous pesticides reduced suicide rates. More recent reviews suggest firearms restrictions may reduce suicides, but with small effect sizes and methodological limitations. Evidence quality ranged from high to critically low (12/20 rated as critically low). With the exception of pesticide restrictions, lower and middle-income settings were not represented.

**Conclusions:**

Several means restriction approaches demonstrate effectiveness, although high study overlap and variable study quality were evident. A focus on differential impacts across sociodemographic groups, more evidence from lower and middle-income countries and evidence for suicide prevention on roads and from residential buildings is needed.

**PROSPERO registration number:**

CRD42024620103.

**WHAT IS ALREADY KNOWN ON THIS TOPIC**

- Multiple systematic reviews of suicide means restriction have been conducted. However, synthesis of their quality, the level of primary study overlap and assessment of the gaps in evidence has not been conducted.

**WHAT THIS STUDY ADDS**

- This study highlights gaps in evidence for suicide means restriction interventions in lower and middle-income countries, except for pesticides restrictions, for impacts across sociodemographic groups and for preventing suicide on roads.
- Strong effect sizes for interventions to prevent jumping from heights should be considered in the context of considerable primary study overlap; in other words, relatively few key sites were included repeatedly.

**HOW THIS STUDY MIGHT AFFECT RESEARCH, PRACTICE OR POLICY**

- Research should focus on equity of prevention efforts by capturing impacts across sociodemographic groups as well as monitoring longer-term impacts of means restriction, including changes to commonly used sites and suicide methods.
- Evidence for means restriction interventions to prevent suicide on roads and from buildings is needed.

## Background

 Means restriction is widely considered to be one of the most effective population-level, evidence-based suicide prevention strategies.^1^ Means restriction interventions tend to require substantial, population-level policy or environmental changes, for example, legislation on medication sales and structural changes to bridges. Due to their scale, interventions tend to focus on commonly used methods of suicide and attempted suicide, or those that are highly lethal. This varies by country and population. For example, studies of restricting access to jumping from bridges have focused on urban populations in high-income countries.^2^ Evidence regarding the impact of firearms restrictions on suicide concentrates around the USA, where more than half of suicide deaths are by firearm.^3^ Much of the evidence on means restriction in low- and middle-income countries has focused on restricting access to highly lethal pesticides in agricultural areas, where pesticide poisoning is a common method of suicide.^4^ Means restriction interventions are frequently assessed in terms of their impact on method-specific suicide attempts and suicide deaths, as well as on overall rates of suicide attempts and suicide deaths. This enables potential method substitution to be captured, whereby restricting one means of suicide inadvertently drives people towards alternative methods.^1^

Two umbrella reviews of suicide prevention measures more broadly found that means restriction demonstrated efficacy on reducing population suicide rates.^5 6^ However, these studies did not include a specific focus on means restriction. Multiple existing systematic reviews (SRs) include several of the same primary studies. Additionally, SRs include multiple primary studies evaluating a single means restrictions site. Existing reviews span over 20 years, and it is not clear where evidence has been superseded by more up-to-date studies.

### Objective

Our objectives were to:

- Review and synthesise the quality and findings of SRs.
- Identify the level of overlap of primary studies within SRs.
- Identify gaps in evidence and identify recent evidence from primary studies to provide additional evidence.

### Study selection and analysis

We conducted an overview of SRs, also known as an umbrella review, following Cochrane guidance^7^ and drawing on other published methodological guidance.^8 9^ Umbrella reviews are appropriate for research questions where a large body of research of multiple interventions exists in the form of SRs. We report findings according to the Preferred Reporting Items for Overviews of Reviews checklist (online supplemental table S1).^10^ The protocol for our study was pre-registered with PROSPERO (CRD42024620103). We did not conduct meta-analysis due to high study overlap (see online supplemental box S1). Minor deviations from study protocol are described in online supplemental box S2.

Included interventions were those that restricted access to means of suicide at the population level. We did not impose age restrictions as it would be expected that all ages would be exposed to the interventions. We excluded interventions implemented within clinical services or specific institutions. Comparison groups were the population exposed to the intervention prior to its implementation or a geographically non-exposed population. The primary outcome was suicide rate by the method targeted by the intervention. Secondary outcomes included overall suicide rates, rates of self-harm/attempted suicide and rates of suicide at nearby sites (to capture method displacement) or from other methods (method substitution).

The primary reviewer (SS) searched Web of Science, Ovid (PsycINFO, EMBASE), Cochrane and PubMed databases. We placed no limit on publication date as at the time of searching no other published umbrella reviews were identified. Our search terms (online supplemental table S2) were developed with an academic librarian. Searches were conducted between 1 October 2024 and 1 March 2025. The second reviewer (SL) screened 20% of titles. Full reference lists of included studies and study protocol databases were also searched (online supplemental box S3). We conducted additional searches for supplemental primary studies using the above search terms, excluding terms relating to ‘systematic review’. We applied a date restriction of January 2020 onwards to account for the lag between studies being published and appearing in SRs. These searches were conducted in April 2025, after data analysis of the included SRs.

Data were extracted using a prespecified proforma based on the research objectives. Review authors were contacted to obtain clarification or additional data if necessary. The quality of the included SRs was assessed by two reviewers using the AMSTAR (A MeaSurement Tool to Assess systematic Reviews-2 guidance^11^ (see online supplemental box S4 for further details).

**Figure 1:**
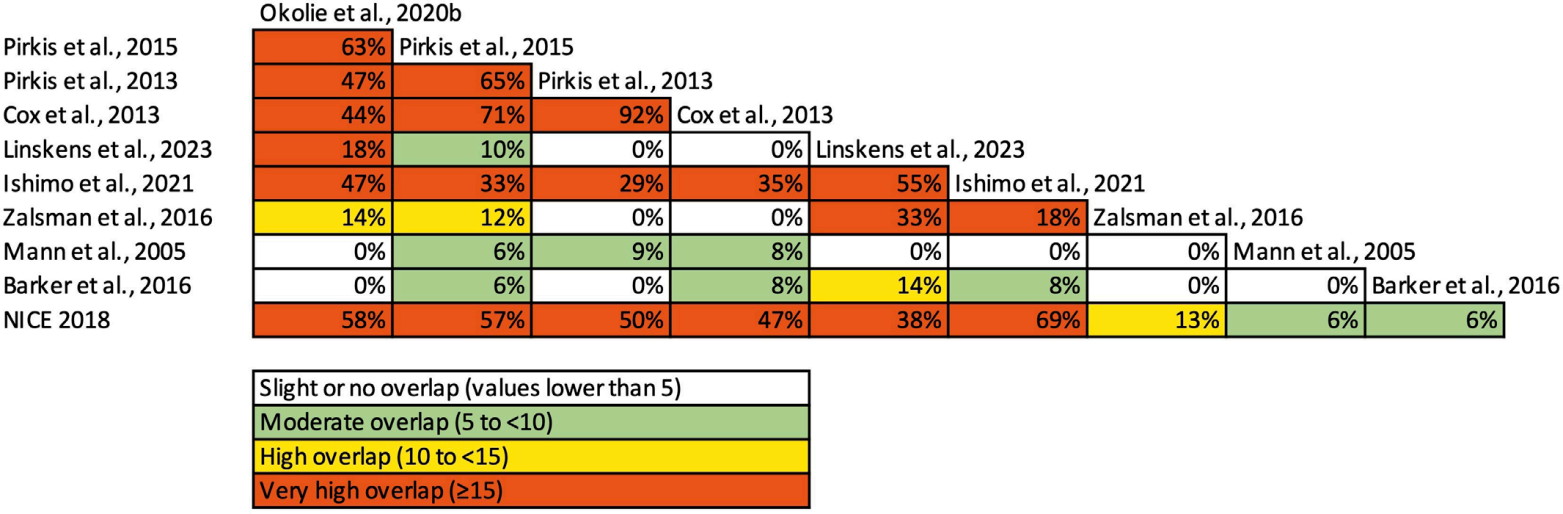
Heat map for primary study overlap analysis between systematic review pairs (n=10 reviews)*.*

**Figure 2:**
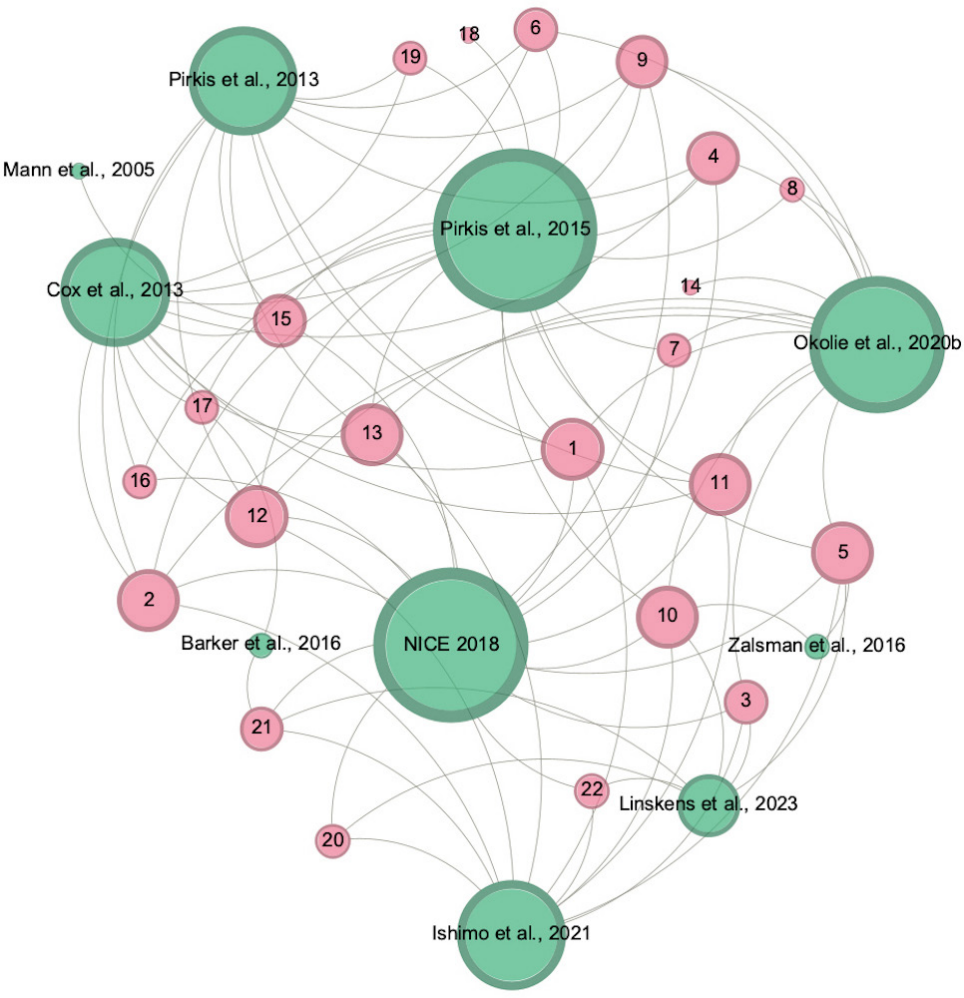
Network diagram for jumping means restriction interventions (N = 10 reviews). Green nodes represent systematic reviews; pink nodes represent primary studies. Larger nodes denote higher numbers of edges. See online supplemental table S4 for primary study ID number and corresponding reference.

We used a Cochrane-recommended evidence-based decision tool to inform our approach to overlapping reviews. ^7^We created a citation matrix and then quantified the degree of overlap by calculating the corrected covered area (CCA).^12^ CCA values can be interpreted as slight overlap (<5), moderate overlap (5–10), high (11-15) or very high (>15). We then mapped the CCAs to each pair of reviews (figure 1 and online supplemental figures S1, S3, S5 and S7).^13^ Due to the relatively high level of overlap in some categories, we prioritised findings from reviews with the highest assessed quality and most recent data, to avoid overstating findings from primary studies included in multiple SRs.^13^ Network diagrams were created using Gephi^14^ to illustrate the specific primary studies included in each SR (figure 2 and online supplemental figures S2, S4, S6 and S8).

The narrative synthesis was centred around the components described by Popay *et al*^15^ which include considering the factors that may explain differences in findings and assessing the robustness of the findings yielded by the synthesis. We grouped studies according to intervention type, country income level and study quality.

## Findings

### Characteristics of included SRs

**Figure 3:**
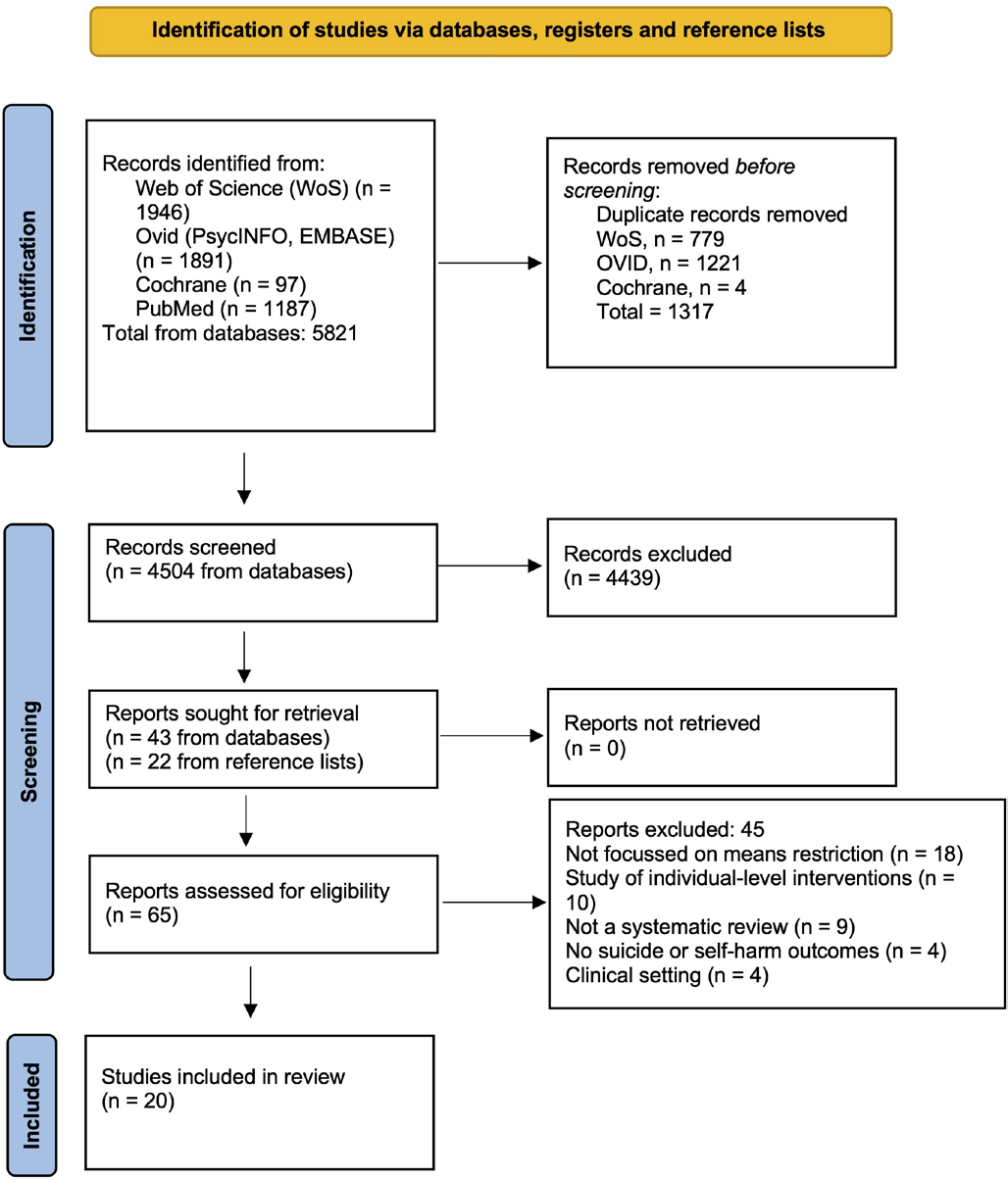
Preferred Reporting Items for Systematic Reviews and Meta-Analyses flow diagram of search.

Our searches returned 5820 titles and 4503 were screened after removing duplicates (figure 3). 65 records were selected for full text screening; 45 were excluded (see list of excluded papers with reasons in online supplemental table S2), and a total of 20 SRs met the study inclusion criteria and were included.

Twenty SRs contained 179 unique primary studies, representing 32 countries (table 1 and online supplemental table S4). We grouped intervention types according to groups used in the SRs, except for railway platform screen doors which we included within the jumping category. The number of reviews included was as follows: jumping (from bridges, viaducts, cliffs and railway platforms), 10 SRs; poisoning by medication, 5; pesticides poisoning, 9; domestic gas, motor vehicle gas and charcoal poisoning, 6; firearms, 6; and roads, 1. Fourteen out of 20 reviews examined displacement to alternative sites or substitution of suicide method. A meta-analysis was conducted in four reviews, all of which focused on interventions to prevent jumping.^216^,^18^

**Table 1:** Summary of included studies (full details in online supplemental table S4)

Author	Category: number and type of interventions included	Summary of study countries and income level	Study outcome measure/s	Main risk estimate from meta-analysis	Events in non-intervention group	Events in intervention group
Okolie *et al*^36^	Roads: no eligible studies	NA	NA	NA	NA	NA
Okolie *et al*^16^	Jumping: 14 studies of means restrictions at bridges and other jumping sites	Switzerland (3) USA (3), UK (2), Canada (2), New Zealand (2) and Australia (2). All high-income countries.	Number of suicide deaths (13 studies), suicide attempts (1)	Suicide deaths: IRR 0.09 (0.03 to 0.27), p<0.001.	742.3 suicide deaths per year	70.6 suicide deaths per year
Pirkis *et al*^2^	Jumping: 9 studies of 8 structural interventions on bridges and cliffs	UK (2), USA (2), New Zealand (2), Canada (1), Switzerland (1). All high-income countries.	Numbers and rates of suicide deaths.	Suicide deaths: RR 0.14, 95% CI 0.09 to 0.21, p=0.001.	436 (5.7 per year)	21 (0.5 per year)
Barker *et al*^21^	Railway: 5 articles of means restriction measures at railway stations.	Hong Kong (2), Japan (1). All high-income countries.	Number of suicide deaths (6), fatality rate of suicide attempts (2), suicide attempts (2).	Meta-analysis was not conducted.	Not available	Not available
Lim *et al*^27^	Poisoning (multiple): 62 studies of pesticides (23 studies), domestic gas (17), motor vehicle exhaust (11) and pharmaceuticals (11).	26 countries: 20 high-income countries (53 studies) and 6 low- and middle-income countries (9 studies).	Change in rate of suicide deaths using the restricted poison, measured using IRR.	Meta-analysis was not conducted.	Not available	Not available
Morgan *et al*^28^	Paracetamol poisoning: 12 studies examining restrictions of paracetamol.	All 12 studies were of the UK (high-income).	Severity of poisoning (8 studies), hospital admission (6) and deaths from paracetamol poisoning (3).	Meta-analysis was not conducted.	Not available	Not available
Gunnell *et al*^4^	Pesticides: 27 studies of specific pesticides bans (12 studies), sales restrictions (8) and mandatory licensing or registering of users (5).	16 countries. 5 low-income or middle-income countries (Bangladesh, Colombia, India, Jordan and Sri Lanka) and 11 high-income (Denmark, Finland, Germany, Greece, Hungary, Ireland, Japan, South Korea, Taiwan, UK and USA).	Rates and numbers of pesticide suicide deaths.	Meta-analysis was not conducted.	Not available	Not available
Reifels *et al*^30^	Pesticides: 5 studies.	3 studies in India and 2 in Sri Lanka. All lower- and middle-income countries.	Rates of suicide (4), suicide from pesticide poisoning (2) and attempted suicide (3).	Meta-analysis was not conducted.	Not available	Not available
Pirkis *et al*^17^	Jumping: 23 articles representing 18 unique studies of jumping.	USA (5), UK (3), NZ (2), Australia (2), Canada (2), Hong Kong (2), Switzerland (2). All high-income countries.	Suicide rates	Suicide deaths: IRR 0.07, 95% CI 0.02 to 0.19, p<0·0001.	863 (5.8 per year)	211 (2.4 per year)
Cox *et al*^22^	Jumping: 9 studies of restricting access to means by installing physical barriers at sites used for jumping.	UK (2), Hong Kong (1), New Zealand (2), USA (2), Switzerland (1), Canada (1). All high-income countries.	Numbers of suicide deaths.	Meta-analysis was not conducted.	Not available	Not available
Linskens *et al*^19^	Multiple: 10 studies of 9 interventions including 4 studies of barriers at bridges or railway stations, 3 of platform screen doors and 3 of restricting access to charcoal.	Hong Kong (2), South Korea (2), Taiwan, Canada, Australia, Switzerland, Japan. All high-income countries.	Suicide deaths (including method-specific) per year.	Meta-analysis was not conducted.	Not available	Not available
Ishimo *et al*^20^	Multiple: 11 studies on physical barriers, 3 studies on pesticides restrictions, 4 studies on domestic gas, motor vehicle gas and charcoal poisoning.	3 studies (of 2 interventions) in Canada, Japan (3 studies), New Zealand (2), Switzerland (2), South Korea (1), UK (1) Australia (1). All high-income countries.	Suicide mortality rates and site/method-specific suicide deaths.	Meta-analysis was not conducted.	Not available	Not available
Zalsman *et al*^23^	Multiple: 24 studies of firearms restrictions (11), medication withdrawal (5), pesticide restriction (5), barriers at jumping sites (2) and charcoal sales restrictions (1).	USA (4), Australia (2), UK (3), Sri Lanka (3), India (1), Norway (1), Sweden (1), Denmark (1), Switzerland (1), Israel (1), New Zealand (1), Austria (1), Canada (1), Hong Kong (1). 4/24 studies in lower- and middle-income countries.	Suicide death, including methods-specific death.	Meta-analysis was not conducted.	Not available	Not available
Mann *et al*^24^	Multiple: 27 studies of barbiturate restrictions (7), firearms restrictions (6), domestic gas detoxification (6), catalytic converters (4), pesticide restrictions (2), barriers to jumping (1), analgesic pack size change (1).	USA (5), Canada (3), Australia (4), Finland (1), Samoa (1), UK (5), Japan (1), New Zealand (1), Germany (1), Norway (1), Sweden (1), Netherlands (1), Denmark (1). One upper-middle income country.	Suicide deaths.	Meta-analysis was not conducted.	Not available	Not available
Mann *et al*^31^	Multiple: 49 studies of firearms restrictions, 2 studies of pesticides, 3 studies of domestic and motor vehicle gas.	List of included studies not available.	Suicide deaths.	Meta-analysis was not conducted.	Not available	Not available
Robinson *et al*^29^	Multiple: 8 studies on means restriction (2 on restriction of SSRIs in under 18 s and 6 on firearms restrictions).	Canada (3), New Zealand (1), Israel (1), Austria (1), UK (1). One study of 23 WHO Stratum A countries.	All suicide deaths, method-specific suicide.	Meta-analysis not conducted.	Not available	Not available
Bailey *et al*^32^	Pesticides (1).	Sri Lanka (1). Lower-middle-income country.	Pesticide self-poisoning.	Meta-analysis was not conducted.	Not available	Not available
Hahn *et al*^34^	Firearms: 15 studies of firearms restrictions.	USA (14) and Canada (2). High income countries.	Suicide deaths.	Meta-analysis was not conducted.	Not available	Not available
National Institute for Health and Care Excellence^18^	Multiple: 16 studies of barriers and safety nets at jumping sites (11), platform screen doors (2), road access restriction (2) and firearm legislation (1).	Canada (2), USA (3), New Zealand (2), Switzerland (1), UK (2), Austria (1), Australia (3), South Korea (1), Japan (1). All high-income countries.	Suicide deaths and suicide attempts.	Suicide deaths: risk ratio=0.24 (95% CI 0.14 to 0.39).	1001 (3.16 per year)	116 (0.72 per year)
Rubbo *et al*^33^	Pesticides: 9 studies of pesticide regulations.	India (2), South Korea (2), Taiwan (2), Japan (1), China (1), Mongolia (1). 4/9 studies were in lower- and middle-income countries.	Suicides by pesticide poisoning.	Meta-analysis was not conducted.	Not available	Not available

IRR, incident rate ratio.

### Jumping

Ten SRs, containing 22 primary studies, evaluated means restriction interventions to prevent jumping. The CCA for interventions aimed at restricting access to jumping was 35% (range 0% to 92%), indicating a very high level of overlap (figures1  2). The quality of the SRs ranged from high^16^ to moderate^19 20^ and critically low (table 2).^217 21^,^24^ The main critical weaknesses that led to studies being downrated from high quality were: the reasons for excluding individual studies not being provided (n=8) and the absence of a pre-registered protocol (n=6).

**Table 2:** Quality of included means restriction systematic reviews (AMSTAR-2)

Intervention type studied	Author	Summary of quality assessment*	Domains of critical weakness and areas of strength
Roads	Okolie *et al*^36^	High	Areas of critical weakness: none The assessment could only be partially completed because the review did not identify any studies meeting the inclusion criteria. Therefore, some parts of the assessment were conducted based on what the study protocol had planned.
Jumping	Okolie *et al*^16^	High	Areas of critical weakness: none.
Jumping	Pirkis *et al*^2^	Critically low † (two areas of critical weakness)	Areas of critical weakness: no preregistered protocol; no reasons given for excluding each potentially relevant study (a summary of reasons for exclusion was provided). Areas of non-critical weakness: no duplication of data extraction and no formal assessment of study quality/risks of bias. Notes: heterogeneity and bias due to study designs and limitations of observational nature of studies were discussed.
Railway	Barker *et al*^21^	Critically low (two areas of critical weakness)	Areas of critical weaknesses: no preregistered protocol, no reasons given for excluding potentially relevant studies. No systematic quality/risk of bias assessments and no duplication of searches or data extraction.
Poisoning	Lim *et al*^27^	Moderate–high	No areas of critical weakness. Areas of non-critical weakness: limited detail on study interventions and settings, limited discussion of publication bias.
Paracetamol poisoning	Morgan *et al*^28^	Critically low † (two areas of critical weakness)	Areas of critical weakness: no preregistered protocol, no reasons given for excluding each potentially relevant study. The study includes discussion of sources of bias arising from study design and heterogeneity. Non-critical weaknesses: no duplication of data extraction and searching and no formal assessment of study quality/risks of bias.
Pesticide poisoning	Gunnell *et al*^4^	Moderate (one area of critical weakness)	Areas of critical weakness: while a summary of reasons for excluding potentially relevant studies was provided, reasons given for excluding each study were not available. Notes: there were several areas indicating a high level of confidence in quality, including a preregistered protocol, risk of bias assessed using Cochrane Effective Practice and Organisation of Care criteria and duplication of data extraction and reviewing.
Pesticide poisoning	Reifels *et al*^30^	Critically low (two areas of critical weakness)	Areas of critical weakness: no preregistered protocol and no clear criteria for/record of excluding potentially relevant studies. Notes: risks of bias were discussed but no systematic quality/risk of bias assessment was conducted.
Jumping	Pirkis *et al*^17^	Critically low (two areas of critical weakness)	Areas of critical weakness: no preregistered protocol and no reasons given for excluding each potentially relevant study (however, a summary of reasons for exclusion was provided). Non-critical weakness: no duplication of data extraction and searching.
Jumping	Cox *et al*^22^	Critically low (two areas of critical weakness)	Areas of critical weakness: no preregistered protocol and no reasons given for excluding potentially relevant studies. Non-critical weaknesses: risks of bias were discussed, though no systematic quality/risk of bias assessment was conducted. No duplication of searches or data extraction.
Multiple: Jumping Domestic gas, motor vehicle gas and charcoal poisoning	Linskens *et al*^19^	Moderate (one area of critical weakness)	Areas of critical weakness: no record of reasons for excluding potentially relevant individual studies. Notes: the study included a preregistered protocol, comprehensive quality assessment and duplication of searches and data extraction. No justification for focussing on a specific time period.
Multiple: Jumping, pesticides, domestic gas, motor vehicle gas and charcoal poisoning	Ishimo *et al*^20^	Moderate (one area of critical weakness)	Areas of critical weakness: no reasons given for excluding individual potentially relevant studies. Notes: study quality was assessed using the Effective Public Health Practice Project Quality Assessment Tool. Searches and data extraction were duplicated and interventions were described in detail.
Multiple: Jumping, pesticides, domestic gas, motor vehicle gas and charcoal poisoning, firearms, medication poisoning	Zalsman *et al*^23^	Critically low (two areas of critical weakness)	Areas of critical weakness: no preregistered protocol, excluded potentially relevant studies, including those rated as ‘very low evidence’ were not listed. Notes: a group of 18 suicide prevention experts worked to reach consensus on the evidence ratings, which studies to include and the study conclusions. The Oxford Centre for Evidence-Based Medicine criteria were used to rate the level of evidence, though this was limited to identifying the study design rather than the quality of the study.
Multiple: Jumping, pesticides, domestic gas, motor vehicle gas and charcoal poisoning, firearms, medication poisoning	Mann *et al*^24^	Critically low † (two areas of critical weakness)	Areas of critical weakness: no preregistered protocol, though the study parameters were designed with a group of 15 suicide experts. Absence of reasons for excluding potentially relevant studies. Notes: The Oxford Centre for Evidence-Based Medicine criteria were used to rate the level of evidence, though this was limited to identifying the study design rather than the quality of the study. The methodological quality and risks of bias of the studies were discussed.
Multiple: pesticides, domestic gas, motor vehicle gas and charcoal poisoning, firearms	Mann *et al*^31^	Critically low (two areas of critical weakness)	Areas of critical weaknesses: no preregistered protocol, though the study parameters were designed with a group of 15 suicide experts, and no list of excluded potentially relevant studies. Notes: the authors focused on RCTs and epidemiological time-series study designs. The methodological quality and risks of bias of the studies were discussed, but no formal assessment was conducted. The list of included means restrictions studies was not provided.
Multiple: Pesticides, firearms, medication poisoning	Robinson *et al*^29^	Critically low (two areas of critical weakness)	Areas of critical weakness: no preregistered protocol and no list of individual potentially relevant studies that were excluded, though summary reasons for exclusion were provided. Notes: duplication of searches and data extraction was conducted.
Pesticide poisoning	Bailey *et al*^32^	Critically low (two areas of critical weakness)	Areas of critical weakness: no preregistration or protocol (though this was an update to Robinson *et al* and methods were based on the original systematic review) and no list of individual potentially relevant studies that were excluded. Notes: risks of bias were assessed by the Cochrane risk of bias tool.
Firearms	Hahn *et al*^34^	Critically low † (two areas of critical weakness)	Areas of critical weakness: no preregistration or protocol, no list of individual potentially relevant studies that were excluded. Notes: a thorough assessment of bias and study quality, and duplication of data extraction and quality assessment was conducted.
Multiple: Jumping, firearms	NICE^18^	Moderate (one area of critical weakness)	Areas of critical weakness: no statistical tests for publication bias in the meta-analysis. Notes: searches were comprehensive, with each excluded study recorded and quality assessments of included studies conducted.
Pesticide poisoning	Rubbo *et al*^33^	High	Areas of critical weakness: none. Notes: The review was preregistered; screening and study assessment were conducted in duplicate; and excluded studies were described. The risks of bias in primary studies were assessed using a Cochrane-recommended modified version of the risk of bias criteria for interrupted time series studies.

* According to the AMSTAR-2 guidance, studies were downgraded to ‘moderate’ if 1 domain of critical weakness was identified, and to ‘critically low’ if 2 or more domains of critical weakness were identified.

† The study was published prior to, or within 2 years of, the launch of PROSPERO and therefore would not be expected to have preregistered a protocol.

AMSTAR-2, A MeaSurement Tool to Assess systematic Reviews-2.

Ten reviews examined the impacts of physical barriers to prevent jumping from high structures such as bridges or cliffs on suicide deaths at those sites. Among reviews rated as high or moderate quality (n=4), all concluded that physical barriers reduced suicide risk at the intervention sites. Two of these conclusions were made with low certainty^19^ or were based on low quality evidence,^16^ with much of the uncertainty arising from the unavoidable observational design of the primary studies. Ishimo *et al*^20^ concluded that physical barriers to prevent suicide were largely effective and that the strength of evidence was ‘moderate to strong’. Effect sizes were generally large, with rate reductions ranging from 53% to 89%, and reported incident rate ratios (IRRs) (comparing before the intervention vs after) of between 0.009 and 0.24, with suicide deaths reducing to zero at some sites. Five reviews rated as ‘critically low’^217 22^,^24^ included conclusions supporting the effectiveness of means restriction for reducing suicides at jumping sites.^17 22 23^

Four reviews included a meta-analysis. One review rated as high quality^16^ had a pooled IRR of 0.09 (0.03 to 0.27, p<0.001; I2=88.40%) from 12 studies of jumping interventions. Another review rated as moderate quality^18^ reported an IRR of 0.2 (95% CI 0.15 to 0.38) from a meta-analysis of 11 studies of physical barriers at jumping sites. Analysis by population subgroups was not conducted. Six SRs synthesised evidence for method substitution.^2 16 18 20 22 24^ Ishimo *et al*^20^ found mixed evidence for substitution when considering all means restriction interventions, while Okolie *et al*^16^ reported that, while evidence for displacement to an alternative jumping site was minimal, further evidence was needed. A meta-analysis of seven studies^18^ found no significant increase in suicides at other sites over a total of 45 years post-intervention follow-up: rate ratio=1.46 (95% CI 0.84 to 2.54) with a moderate level of certainty in the evidence.

Six SRs included studies of railway platform screen doors,^17^,^22^ though only two^18 21^ reported evidence specifically for this intervention. Both studies reported ‘strong evidence’ in support of platform screen doors in preventing site-specific suicide deaths, although NICE^18^ concluded that more research was needed due to the evidence arising from single railway networks. Ishimo^20^ included two studies of platform screen doors, examining their effectiveness alongside other physical barriers to prevent jumping, and concluded that physical barriers were effective at reducing site-specific mortality. The primary studies were rated as moderate to high quality. There was no reported evidence of displacement to other railway sites. Half-height barriers were deemed less effective than full-height barriers. Most intervention sites across reviews included underground railway systems (n=3), though one review did not specify. Examining bridges and railway barriers together, Linskens found low certainty evidence that barriers may reduce suicide deaths.^19^

Six SRs examined the impact of restricting road access to a jumping site. 216,18 20 22However, as only two primary studies^25 26^ were included across the SRs, discrete synthesis of this specific intervention was limited.

Two SRs^20 22^ examined differential effects by sex. However, within these, only one primary study reported results by sex, finding evidence for a reduction in suicide for men but not women, following construction of a bridge barrier.

### Poisoning by medication

Five SRs (including 33 primary studies) examined restricting access to medication to reduce suicide deaths from poisoning. 2324 27,29One review was assessed as moderate to high quality^27^ and four as critically low. ^23 24 28 29^The two areas of critical weaknesses were: no preregistered protocol (n=4) and no reasons given for excluding each potentially relevant study (n=5). The CCA for SRs was 4%, indicating slight overlap (online supplemental figure S5 and S6).

Fifteen SRs (of 12 separate interventions) examined the impact of restricting availability of over-the-counter paracetamol, with the majority (12 primary studies) included in only one of the SRs. ^28^This intervention type was the only to examine non-fatal self-harm as an outcome as well as death by suicide. Morgan *et al* concluded that hospital admissions for self-poisoning appear to have reduced following paracetamol pack size regulations in 1998 in the UK, with reductions of between 11% and 31% found in five out of six studies. Evidence concerning impacts on fatal intentional self-poisoning was mixed, and Morgan *et al* concluded that restricting paracetamol availability may not be sufficient as a standalone measure. Limitations of the primary studies included relatively short follow-up periods of 1 to 2 years and heterogeneity of comparison groups. However, subsequent studies, as reviewed in Zalsman *et al*,^23^ found evidence that pack size restrictions were linked to reductions in deaths and self-poisoning from paracetamol.

Lim *et al*^27^ included 11 studies on restricting access to medications commonly used for self-poisoning (including paracetamol and salicylates) and those with high lethality when used in poisoning (dextropropoxyphene, barbiturates and caffeine tablets) and concluded that the restrictions were associated with decreases in suicide by poisoning. The authors found no evidence of method substitution when they examined suicide rates overall and by poisoning with other substances. All primary studies were conducted in high-income countries, with most assessed as having low or medium risks of bias.

Robinson *et al*^29^ found no evidence that restricting the use of prescribed SSRIs among young people (aged under 20 years) reduced overall rates of suicide or self-harm hospitalisations. Findings were based on two primary studies including one multinational study conducted in 23 countries.

### Pesticide poisoning

Nine SRs, including 47 primary studies, examined policies and interventions aimed at restricting access to highly hazardous pesticides.^420 23 24 27 30^,^32^ One review was rated high quality,^33^ one was rated as moderate to high quality,^27^ two as moderate^4 20^ and five as critically low.^2324 30^,^32^ There was moderate primary study overlap across the SRs (CCA 8%) (online supplemental figures S1 and S2).

Lim *et al*^27^ included 23 studies of pesticide restrictions in 15 countries, including five lower- and middle-income countries. Most studies had low or medium risks of bias. Decreases in suicides from pesticide poisoning were reported in 19/23 studies. Where IRRs could be estimated, they ranged from 0.37 to 0.69. The review concluded that pesticide bans were more effective than licensing requirements. Increases in suicide deaths by other methods did not generally occur, though an increase in suicide by hanging was observed in India and some longer-term increases were observed in Taiwan following pesticide bans. Gunnell *et al*^4^ included 15 of the same studies as Lim *et al*, plus 12 additional ones. Countries included five LMICs and 11 HICs. The authors concluded that pesticide bans were associated with reductions in suicide from pesticide poisoning as well as lower overall suicide rates. In an update to Gunnell *et al*, Rubbo *et al* synthesised studies published from 2017 and found strong evidence, including from methods accounting for pre-existing trends, that pesticide bans support reductions in pesticide and overall suicide rates.^33^ Reductions in pesticide suicide rates reported in higher quality studies were in the region of 28%–61%, while reductions in overall suicide rates ranged from 7% to 45%.

Findings related to non-fatal self-poisoning were synthesised in one of the reviews.^30^ Reifels *et al* focused on regulations that reduced access to pesticides, rather than banning them.^30^ From five included studies, only one was assessed as being sufficiently powered and found no evidence that self-poisoning by pesticide was reduced.

Other SRs included relatively low numbers of primary studies of pesticide regulations, with such interventions studied as part of a broader synthesis of suicide prevention initiatives.^20 23 24 31 32^ The conclusions reached were broadly similar to Lim *et al* and Gunnell *et al*.

### Domestic gas, motor vehicle gas and charcoal

Six SRs^19 20 23 24 27 31^ including a total of 37 primary studies examined restrictions on availability of toxic gas, including detoxification of domestic gas supplies (18 studies of nine high-income countries), the introduction of catalytic converters to motor vehicles (17 studies of six high-income countries) (one study examined both) and restrictions on the availability of charcoal (three studies of three high-income countries). The reviews were rated as moderate to high (n=1),^27^ moderate (2)^19 20^ and critically low (3).^23 24 31^ The degree of primary study overlap across SRs (CCA) was moderate at 5% (online supplemental figures S3 and S4).

Most of the primary studies (n=25) were included in one moderate to high quality SR;^27^ therefore, we prioritised this synthesis in our overview. Lim *et al* found reduced incidence of suicide by domestic gas poisoning in all studies in which an IRR could be calculated, with IRRs ranging from 0.03 to 0.82 (13 countries). Suicide deaths by other methods increased in eight countries (IRR range 1.24 to 1.66), decreased in two (IRR 0.78 and 0.87) and did not change in three. IRRs for suicide by motor vehicle exhausts were calculated for eight countries, with decreases found in five countries and increases in two. However, while suicide incidence by other methods subsequently decreased in three counties, increases were observed in two.

Linskens *et al*^19^ included three studies of regulations to restrict the purchase of charcoal. While all three studies reported reductions in suicide deaths by charcoal burning poisoning post-intervention, the certainty of the evidence was rated as low.

### Firearms

Six SRs, including a total of 38 primary studies, included synthesis of restrictions on the availability of firearms.^23 24 29 31 34^ One SR was assessed as being of moderate quality, and five were rated as critically low. The level of overlap within the SRs was 4%, which is considered low (online supplemental figures S7 and S8).

Hahn *et al*^34^ examined bans on specific firearms or ammunition, restrictions on acquisition or possession, licensing of firearms, ‘shall issue’ laws, storage laws and combinations of laws. The review of 15 studies concluded that studies were of limited quality to determine whether laws were associated with suicide rates. All included studies were US-based and the review was conducted over 20 years ago. Mann *et al*^31^ referred to 49 studies of firearms restriction but did not include a list of these studies (our request to obtain the list of included studies was unsuccessful). A recently published SR (identified as part of our search for up-to-date evidence) by Shank *et al*^35^ included 27 studies on firearms (25 from the USA and two from Australia). While the authors concluded that firearms interventions were associated with reduced suicide deaths, significant methodological limitations and small effects sizes were noted.

Robinson *et al*,^29^ in their review of youth suicide prevention, examined six studies and found reductions in the firearm suicide rate following policies designed to restrict firearms access in five studies, with one reporting an increase. However, overall suicide rates did not decrease, which the authors concluded may be due to firearms being a relatively uncommon method of suicide in young people in the countries studied. The NICE review^18^ included one study examining suicide rates in US states that required background checks or mandatory waiting periods to acquire a handgun. States with such laws had lower firearm suicide rates compared with states without such laws, although the level of certainty in the evidence was very low due to the observational study design and potential effects of other suicide prevention initiatives.

### Roads

Our search identified one SR on means restriction to prevent suicide on roads.^36^ However, this Cochrane review included no published primary studies. Subsequent searches for relevant studies published after those identified by Okolie *et al* resulted in no new primary studies found.

## Conclusions and clinical implications

### Main findings

Interventions aimed at reducing suicide deaths from jumping at specific sites are likely to be effective at reducing site-specific and overall suicide rates. Effect sizes were considerable, though evidence for displacement of deaths to other sites was found. Substantial primary study overlap was evident. Medication pack size restrictions, pharmacy only sales and medication bans were associated with reduced hospital admissions and suicide deaths from medication-specific poisonings, though longer follow-up would clarify the enduring impacts. Pesticide bans appear to be the most effective form of pesticide restriction, with reductions in both pesticide suicides and overall suicide deaths observed. Detoxification of domestic gas supplies and motor vehicle exhausts was associated with reduced suicide deaths by these methods, though there was evidence of method substitution. Evidence for firearms restrictions is limited to small effect sizes and is hampered by the quality of the evidence. With the exception of SRs of pesticide restrictions (n=9), most reviews (n=11/12) comprised studies from high-income countries only.

### Strengths and limitations

We conducted a comprehensive umbrella review to identify SRs of suicide means restriction. Screening, data extraction and study quality assessment were duplicated to optimise accuracy and reliability. We designed an inclusive search strategy and screened over 5800 titles. We conducted a narrative synthesis of the reviews based on prespecified groupings to maximise transparency. While we assessed the quality of the SRs and incorporated the authors’ quality assessment of the primary studies (where available) into our synthesis, we did not undertake our own quality assessments of the 179 primary studies. Individual primary study methodological limitations are likely to be widespread due to the observational nature of most of the primary study designs, although we reflected such limitations in our synthesis.

### Comparison with existing evidence

A recent umbrella review by Nevarez-Flores *et al*^37^ identified 12 SRs of suicide means restriction. The authors concluded that means restriction focused on restricting jumping from heights and in front of moving objects, access to firearms and substances used in self-poisoning should be recommended for suicide prevention. There were several differences between Nevarez-Flores *et al*’s review and ours. First, their conclusions were based on twelve studies, while our search identified twenty. Second, we considered the level of primary study overlap and the potential impact of overlap on the conclusions (a vital component of review overviews). Third, we included a search for up-to-date primary studies published after the cut-off for the SR searches. Finally, our narrative synthesis incorporated a specific focus on prioritising findings from higher quality SRs while also considering the specific components contributing to reviews being rated as lower quality. We highlight the areas where more and higher quality evidence is needed, namely for suicide on roads and from buildings, firearms restrictions, for differential effects in sociodemographic groups and for interventions (not related to pesticides) in low- and middle-income countries.

### Implications

Findings regarding displacement of suicide site or method substitution were heterogeneous, depending on the intervention type. Pirkis *et al*^2^ found a substantial (44%) increase in jumping suicides per year at sites near to the intervention site. While this was offset by an 86% reduction at the intervention sites, this finding highlights the potential scope of displacement of suicide to alternative sites and the need for continued monitoring. Regarding poisoning-related suicide deaths, Lim *et al* found no overall evidence for an increase in suicides by alternative methods following pesticide, gas and medicines restrictions.

Few studies considered differential impacts on sociodemographic groups. Two reviews^20 22^ examined effects by sex, although only one primary study reported findings for males and females separately. Interventions targeting lethal means of suicide such as jumping from a height are likely to have a greater effect on male suicide.^38^ Conversely, rates of medication self-poisoning are higher among females;^39^ therefore, interventions focussing on availability of commonly-used medications may have greater impact on female suicide rates. The reported reductions in non-fatal self-poisoning by paracetamol, following sales restrictions, is especially pertinent for women, who comprise more than eight out of ten patients presenting to hospital following intentional paracetamol poisoning in the UK.^40^

While the evidence for restricting access to sites used for jumping is relatively strong for high-income countries, there is no evidence from low- and middle-income countries. In addition, there was significant primary study overlap in the SRs of restricting access to suicide by jumping, which should be considered when interpreting results. There are also other factors to consider when implementing such interventions. Hemmer *et al* concluded that full barriers were more effective than partial barriers and that those of at least 2.3 metres in height, and inbound barriers, were most effective.^41^ Accompanying activities such as responsible media reporting of suicide deaths, for example, those at frequently used locations or celebrity deaths, can support jumping restrictions interventions.^17 42 43^

Our included reviews found evidence to support restrictions in the availability of paracetamol. However, paracetamol remains a common medication used in self-poisoning, used in around a third of hospital-presenting episodes in the UK.^44^ Therefore, consideration of further restrictions is warranted. In 2018, codeine (including paracetamol-codeine combinations) was restricted to prescription-only in Australia. A subsequent study linked the intervention with reduced numbers of hospital-treated poisonings for paracetamol-codeine combinations.

The small effect sizes (and lower quality of evidence) concerning restricting access to firearms may be due to inadequate data on gun ownership.^34^ In addition, in communities where access to firearms is widespread, households may have access to multiple guns for a range of purposes, potentially hampering restriction efforts.

The umbrella review has highlighted several areas of weaknesses in the design of SRs. The absence of comprehensive quality assessment of primary studies impacted our ability to assess the evidence. Few SRs included preregistered protocols. However, this largely reflects higher expectations of preregistration since 2011 when PROSPERO was introduced.^45^ Studies omitting the reasons for excluding primary studies at the full text stage reduced the transparency of review processes.

We propose several implications for future research. The absence of studies evaluating means restriction for preventing suicides on roads is notable. Such interventions could include structures to prevent access to roads, strategies aimed at increasing the likelihood of human intervention (eg, camera surveillance) and encouraging help-seeking at the site.^36^ There is also a relative lack of evidence concerning suicide means restriction for preventing jumping from residential buildings and car parks. Two-thirds of the SRs incorporated some analysis of location displacement, method substitution or other unintended consequences, for example, by measuring overall suicide rates. However, several reviews noted limitations of the primary studies in assessing such unintended outcomes. Future studies should focus on longer-term impacts of means restriction to capture gradual shifts to alternative methods.

Emerging approaches for means restriction include designs that incorporate well-being and avoid drawing attention to suicide methods.^46^ Video or sensor-based surveillance and spinning rollers on bridge barriers to prevent climbing have recently been evaluated,^47^ with spinning rollers found to be effective. Interventions should be developed with people with lived experiences of suicidal behaviour, who can provide invaluable insights into the design, acceptability and potential mechanisms for the effect of interventions.^48^

### Conclusion

Means restriction is a key, evidence-based suicide prevention strategy. Greater focus on differential impacts across sociodemographic groups is needed. Additionally, more evidence from lower and middle-income countries and evidence for suicide prevention on roads and from residential buildings would address knowledge gaps.

## Supplementary material

10.1136/bmjment-2025-302069 online supplemental file 1

## Data Availability

Data are available upon reasonable request.
