# Supplementary material for: Effectiveness of suicide means restriction: an overview of systematic reviews
Source: BMJ Ment Health. 2025 Dec 9;28(1):e302069. doi: 10.1136/bmjment-2025-302069 (PMC12699607; doi:10.1136/bmjment-2025-302069)
Supplement: online supplemental file 1 [file bmjment-28-1-s001.docx]

**Supplementary material**

**Table S1**: PRIOR checklist [10]

| Section Topic | # | Item | Location reported |
| --- | --- | --- | --- |
| TITLE | |  |  |
| Title | 1 | Identify the report as an overview of reviews. | Title, page 1 |
| ABSTRACT | |  |  |
| Abstract | 2 | Provide a comprehensive and accurate summary of the purpose, methods, and results of the overview of reviews. | Abstract, page 1 |
| INTRODUCTION | |  |  |
| Rationale | 3 | Describe the rationale for conducting the overview of reviews in the context of existing knowledge. | Background, paragraphs 1-2, page 3 |
| Objectives | 4 | Provide an explicit statement of the objective(s) or question(s) addressed by the overview of reviews. | Background, paragraph 3, page 3 |
| METHODS | |  |  |
| Eligibility criteria | 5a | Specify the inclusion and exclusion criteria for the overview of reviews. If supplemental primary studies were included, this should be stated, with a rationale. | Study selection and analysis, paragraphs 1-2, page 4 |
|  | 5b | Specify the definition of ‘systematic review’ as used in the inclusion criteria for the overview of reviews. | PROSPERO protocol, Section 22 |
| Information sources | 6 | Specify all databases, registers, websites, organizations, reference lists, and other sources searched or consulted to identify systematic reviews and supplemental primary studies (if included).  Specify the date when each source was last searched or consulted. | Study selection and analysis, paragraphs 1-3, page 4 |
| Search strategy | 7 | Present the full search strategies for all databases, registers and websites, such that they could be reproduced. Describe any search filters and limits applied. | Supplement (Table S2) and Study selection and analysis, paragraph 3, page 4 |
| Selection process | 8a | Describe the methods used to decide whether a systematic review or supplemental primary study (if included) met the inclusion criteria of the overview of reviews. | Study selection and analysis, paragraph 2, page 4 |
|  | 8b | Describe how overlap in the populations, interventions, comparators, and/or outcomes of systematic reviews was identified and managed during study selection. | Study selection and analysis, paragraph 5, page 4 and Supplement (Box S3) |
| Data collection process | 9a | Describe the methods used to collect data from reports. | Study selection and analysis, paragraph 4, page 4 |
|  | 9b | If applicable, describe the methods used to identify and manage primary study overlap at the level of the comparison and outcome during data collection. For each outcome, specify the method used to illustrate and/or quantify the degree of primary study overlap across systematic reviews. | Study selection and analysis, paragraph 5, page 4 |
|  | 9c | If applicable, specify the methods used to manage discrepant data across systematic reviews during data collection. | Study selection and analysis, paragraph 4, page 4 |
| Data items | 10 | List and define all variables and outcomes for which data were sought. Describe any assumptions made and/or measures taken to identify and clarify missing or unclear information. | Table 1, page 6 |
| Risk of bias assessment | 11a | Describe the methods used to *assess* risk of bias or methodological quality of the included systematic reviews. | Supplement (Box S4) and Study selection and analysis paragraph 4, page 4 |
|  | 11b | Describe the methods used to *collect* data on (from the systematic reviews) and/or *assess* the risk of bias of the primary studies included in the systematic reviews. Provide a justification for instances where flawed, incomplete, or missing assessments are identified but not re-assessed. | Strengths and limitations, page 25 |
|  | 11c | Describe the methods used to *assess* the risk of bias of supplemental primary studies (if included). |  |
| Synthesis methods | 12a | Describe the methods used to summarize or synthesize results and provide a rationale for the choice(s). | Study selection and analysis, paragraph 6, page 4 |
|  | 12b | Describe any methods used to explore possible causes of heterogeneity among results. | Study selection and analysis, paragraph 6, page 4 |
|  | 12c | Describe any sensitivity analyses conducted to assess the robustness of the synthesized results. | Study selection and analysis |
| Reporting bias assessment | 13 | Describe the methods used to *collect* data on (from the systematic reviews) and/or *assess* the risk of bias due to missing results in a summary or synthesis (arising from reporting biases at the levels of the systematic reviews, primary studies, and supplemental primary studies, if included). | N/A |
| Certainty assessment | 14 | Describe the methods used to *collect* data on (from the systematic reviews) and/or *assess* certainty (or confidence) in the body of evidence for an outcome. | Study selection and analysis, paragraph 6, page 4 |
| RESULTS | |  |  |
| Systematic review and supplemental primary study selection | 15a | Describe the results of the search and selection process, including the number of records screened, assessed for eligibility, and included in the overview of reviews, ideally with a flow diagram. | Findings, |
|  | 15b | Provide a list of studies that might appear to meet the inclusion criteria, but were excluded, with the main reason for exclusion. | Supplement, Table S3 |

| Section Topic | # | Item | Location reported |
| --- | --- | --- | --- |
| Characteristics of systematic reviews and supplemental primary studies | 16 | Cite each included systematic review and supplemental primary study (if included) and present its characteristics. | Table 1, page 6-16 |
| Primary study overlap | 17 | Describe the extent of primary study overlap across the included systematic reviews. | Findings, page 21-24, Figure 2 and Supplementary Figures S1-S4 |
| Risk of bias in systematic reviews, primary studies, and supplemental primary studies | 18a | Present assessments of risk of bias or methodological quality for each included systematic review. | Table 2, page 20 |
|  | 18b | Present assessments (*collected* from systematic reviews or *assessed* anew) of the risk of bias of the primary studies included in the systematic reviews. | Table 1, page 6-16 |
|  | 18c | Present assessments of the risk of bias of supplemental primary studies (if included). | Findings, page 21-24 |
| Summary or synthesis of results | 19a | For all outcomes, summarize the evidence from the systematic reviews and supplemental primary studies (if included). If meta-analyses were done, present for each the summary estimate and its precision and measures of statistical heterogeneity. If comparing groups, describe the direction of the effect. | Findings, page 21-24 |
|  | 19b | If meta-analyses were done, present results of all investigations of possible causes of heterogeneity. | N/A |
|  | 19c | If meta-analyses were done, present results of all sensitivity analyses conducted to assess the robustness of synthesized results. | N/A |
| Reporting biases | 20 | Present assessments (*collected* from systematic reviews and/or *assessed* anew) of the risk of bias due to missing primary studies, analyses, or results in a summary or synthesis (arising from reporting biases at the levels of the systematic reviews, primary studies, and supplemental primary studies, if included) for each summary or synthesis assessed. | Table 2, page 20, and Findings page 21-24 |
| Certainty of evidence | 21 | Present assessments (*collected* or *assessed* anew) of certainty (or confidence) in the body of evidence for each outcome. | Findings page 21-24 |
| DISCUSSION | | |  |
| Discussion | 22a | Summarize the main findings, including any discrepancies in findings across the included systematic reviews and supplemental primary studies (if included). | Conclusions and clinical implications, ‘Main findings’, page 25 |
|  | 22b | Provide a general interpretation of the results in the context of other evidence. | ‘Comparison with existing evidence’, page 25-26 |
|  | 22c | Discuss any limitations of the evidence from systematic reviews, their primary studies, and supplemental primary studies (if included) included in the overview of reviews. Discuss any limitations of the overview of reviews methods used. | ‘Strengths and limitations’, page 25 |
|  | 22d | Discuss implications for practice, policy, and future research (both systematic reviews and primary research). Consider the relevance of the findings to the end users of the overview of reviews, e.g., healthcare providers, policymakers, patients, among others. | ‘Implications’, page 26-27 |
| OTHER INFORMATION | | |  |
| Registration and protocol | 23a | Provide registration information for the overview of reviews, including register name and registration number, or state that the overview of reviews was not registered. | Abstract, page 1, and Study selection and analysis, paragraph 1, page 3 |
|  | 23b | Indicate where the overview of reviews protocol can be accessed, or state that a protocol was not prepared. | Abstract, page 1, and Study selection and analysis, paragraph 1, page 3 |
|  | 23c | Describe and explain any amendments to information provided at registration or in the protocol. Indicate the stage of the overview of reviews at which amendments were made. | Supplement, Box S2 and Study selection and analysis, paragraph 1, page 3 |
| Support | 24 | Describe sources of financial or non-financial support for the overview of reviews, and the role of the funders or sponsors in the overview of reviews. | Funding, page 27-28 |
| Competing  interests | 25 | Declare any competing interests of the overview of reviews' authors. | Competing interests, page 28 |
| Author information | 26a | Provide contact information for the corresponding author. | Title page, page 1 |
|  | 26b | Describe the contributions of individual authors and identify the guarantor of the overview of reviews. | Author contributions, page 28 |
| Availability of data and other materials | 27 | Report which of the following are available, where they can be found, and under which conditions they may be accessed: template data collection forms; data collected from included systematic reviews and supplemental primary studies; analytic code; any other materials used in the overview of reviews. | N/A |

**Table S2**: Example search terms

Web of Science:

TI=((systematic review OR meta-analysis) AND (restrict* OR limit* OR access* OR legisl* OR warning* OR label* OR barrier* OR storage* OR storing* OR means OR rail* OR road OR traffic OR method OR intervention* OR firearm*  OR shoot* OR armoury or armouries or arsenal OR fire arm* OR gun* OR handgun* OR pistol* OR revolver* OR rifle* OR shoot* OR lethal mean* OR lethal weapon* OR ammunition* OR bullet*) AND (tablet OR paracetamol OR analgaesi* OR analgesi* OR drug* OR medic* OR pois* OR overdos* OR self-harm OR suic*))

**Box S1**: Meta-analysis

We aimed to conduct meta-analysis (MA) for interventions where we found two or more systematic reviews with MA for the same category of means restriction. Four systematic reviews, of one means restriction category, met the criteria for MA. We planned to use a Poisson regression model with random intervention effects. [1] This Cochrane-recommended method produces estimates with low levels of bias in instances where the number of studies in a meta-analysis is low and where some control or intervention periods have zero events (as was the case in the present review). This method accounts for baseline variability in suicide rates and for between-study heterogeneity in the intervention effect.

**Box S2**: Differences from protocol

We added the following terms to our search strategy after registering our protocol due to our original search not capturing key means restriction interventions: ‘firearm* OR shoot* OR armour* OR arsenal or fire arm* OR gun* OR handgun* OR pistol* OR revolver* OR rifle* OR shoot* OR lethal mean* OR lethal weapon* OR ammunition* OR bullet*’. The primary reviewer (SS) and the secondary reviewer (SL) both conducted 100% of the AMSTAR-2 quality assessments, rather than the secondary reviewer assessing 20%. In addition, the authorship team discussed the AMSTAR-2 ratings to reach consensus about which components were deemed to be critical flaws.

**Box S3:** Additional search details

We searched Cochrane and PROSPERO databases for protocols for umbrella reviews of suicide means restriction. No Cochrane protocols or reviews were identified. One potentially overlapping PROSPERO protocol was identified: (CRD42022343503) We contacted the protocol’s authors and established that their work on the means restriction component of their project was unlikely to progress within the planned timescale of our review. Therefore, we proceeded with our umbrella review.

**Box S4**: AMSTAR-2 study assessments procedure

Each domain was independently assessed by two authors (SS and SL) and a judgement about the overall confidence in the review was made based on the number of weaknesses found and how critical their impact on the review quality was considered to be [11]. Disagreements were discussed with a third reviewer (LQ). The independent overall ratings were the same for the two reviewers in 17 out of 19 SRs. We considered the seven critical domains and deemed them all to be critically important in the assessment of included systematic reviews, except those relating to meta-analysis (MA) when MA was not conducted. For other non-critical domains, we made a judgement based on the context of the review. For example, most systematic reviews did not report the funding sources for the individual studies included. However, the impact of this was judged to be minimal as means restriction interventions are unlikely to be funded by commercial industry. In summary, we had high confidence in the reviews where we found no or one non-critical weakness. Where one critical weakness was found, we rated the review as moderate and if there was were two or more critical flaws the level of confidence was rated as critically low, as proposed in AMSTAR-2 guidance.

**Table S3**: Excluded studies

|  | Reason code | Source |
| --- | --- | --- |
| 1 | Does not include suicide or self-harm as an outcome | Clinician Attitudes, Screening Practices, and Interventions to Reduce Firearm-Related Injury. 2016 Roszko P.J.D. Ameli J., Carter P.M., Cunningham R.M., Ranney M.L.Epidemiologic Reviews. 38(1) (pp 87-110), 2016. |
| 2 | Does not include suicide or self-harm as an outcome | Homicide-suicides compared to homicides and suicides: Systematic review and meta-analysis. 2013 Panczak R., Geissbuhler M., Zwahlen M., Killias M., Tal K., Egger M. Forensic Science International. 233(1-3) (pp 28-36), 2013. |
| 3 | Not focussed on means restriction | A systematic review of road traffic suicides: Do we know enough to propose effective preventive measures? Radun, Igor; Kannan, Pragathy; Partonen, Timo; Hawton, Keith |
| 4 | Does not include suicide or self-harm as an outcome | R. Ryan, N. Santesso, D. Lowe, S. Hill, J. Grimshaw, M. Prictor, C. Kaufman, G. Cowie and M. Taylor 4 Interventions to improve safe and effective medicines use by consumers: an overview of systematic reviews COCHRANE DATABASE OF SYSTEMATIC REVIEWS |
| 5 | Study of individual-level interventions | C. S. Harrod, C. W. Goss, L. Stallones and C. DiGuiseppi 2014 Interventions for primary prevention of suicide in university and other post‐secondary educational settings Cochrane Database of Systematic Reviews |
| 6 | Study of individual-level interventions | K. L. Gore, C. Chen, N. Fu, J. Larkin, A. Motala and S. Hempel 4 Interventions for People Who Have Attempted Suicide and Their Family Members: A Systematic Review. Rand health quarterly 10 Interventions for People Who Have Attempted Suicide and Their Family Members: A Systematic Review. |
| 7 | Study of individual-level interventions | P. H. Joo and B. J. Yee 4 Efficient Suicide Prevention Intervention: Meta-Analysis Journal of Korean Academy of Psychiatric and Mental Health Nursing 22 Efficient Suicide Prevention Intervention: Meta-Analysis |
| 8 | Study of individual-level interventions | I. Feldman, M. Gebreslassie, F. Sampaio, C. Nystrand and R. Ssegonja 2 Economic Evaluations of Public Health Interventions to Improve Mental Health and Prevent Suicidal Thoughts and Behaviours: A Systematic Literature Review ADMINISTRATION AND POLICY IN MENTAL HEALTH AND MENTAL HEALTH SERVICES RESEARCH 48 |
| 9 | Does not include suicide or self-harm as an outcome | V. M. Constantino, B. M. Fregonesi, K. A. de Abreu Tonani, G. S. Zagui, A. P. Contiero Toninato, E. R. dos Santos Nonose, L. A. Fabriz and S. I. Segura-Munoz 2 Storage and disposal of pharmaceuticals at home: a systematic review CIENCIA & SAUDE COLETIVA 25 Storage and disposal of pharmaceuticals at home: a systematic review |
| 10 | Not focussed on means restriction | R. Benson, J. Rigby, C. Brunsdon, G. Cully, L. S. Too and E. Arensman 9 Quantitative Methods to Detect Suicide and Self-Harm Clusters: A Systematic Review INTERNATIONAL JOURNAL OF ENVIRONMENTAL RESEARCH AND PUBLIC HEALTH 19 Quantitative Methods to Detect Suicide and Self-Harm Clusters: A Systematic Review |
| 11 | Not focussed on means restriction | N. Morovatdar, M. Moradi-Lakeh, S. K. Malakouti and M. Nojomi 4 Most Common Methods of Suicide in Eastern Mediterranean Region of WHO: A Systematic Review and Meta-Analysis ARCHIVES OF SUICIDE RESEARCH 17 Most Common Methods of Suicide in Eastern Mediterranean Region of WHO: A Systematic Review and Meta-Analysis |
| 12 | Not focussed on means restriction | Z. Cai, A. Junus, Q. Chang and P. S. F. Yip The lethality of suicide methods: A systematic review and meta-analysis JOURNAL OF AFFECTIVE DISORDERS 300 The lethality of suicide methods: A systematic review and meta-analysis |
| 13 | Study of individual-level interventions | M. J. Crawford, O. Thomas, N. Khan and E. Kulinskaya Psychosocial interventions following self-harm - Systematic review of their efficacy in preventing suicide BRITISH JOURNAL OF PSYCHIATRY 190 Psychosocial interventions following self-harm - Systematic review of their efficacy in preventing suicide |
| 14 | Study of individual-level interventions | Hunter AA, DiVietro S, Boyer M, Burnham K, Chenard D, Rogers SC. The practice of lethal means restriction counseling in US emergency departments to reduce suicide risk: a systematic review of the literature. Inj Epidemiol. 2021 Sep 13;8(Suppl 1):54. doi: 10.1186/s40621-021-00347-5. |
| 15 | Not focussed on means restriction | Witt K, Chitty KM, Wardhani R, Värnik A, de Leo D, Kõlves K. Effect of alcohol interventions on suicidal ideation and behaviour: A systematic review and meta-analysis. Drug Alcohol Depend. 2021 Sep 1;226:108885. doi: 10.1016/j.drugalcdep.2021.108885. |
| 16 | Clinical setting | Ferguson M, Rhodes K, Loughhead M, McIntyre H, Procter N. The Effectiveness of the Safety Planning Intervention for Adults Experiencing Suicide-Related Distress: A Systematic Review. Arch Suicide Res. 2022 Jul-Sep;26(3):1022-1045. doi: 10.1080/13811118.2021.1915217. |
| 17 | Study of individual-level interventions | Fox KR, Huang X, Guzmán EM, Funsch KM, Cha CB, Ribeiro JD, Franklin JC. Interventions for suicide and self-injury: A meta-analysis of randomized controlled trials across nearly 50 years of research. Psychol Bull. 2020 Dec;146(12):1117-1145. doi: 10.1037/bul0000305. |
| 18 | Not focussed on means restriction | Leske S, Paul E, Gibson M, Little B, Wenitong M, Kolves K. Global systematic review of the effects of suicide prevention interventions in Indigenous peoples. J Epidemiol Community Health. 2020 Dec;74(12):1050-1055. doi: 10.1136/jech-2019-212368. |
| 19 | Clinical setting | Doupnik SK, Rudd B, Schmutte T, Worsley D, Bowden CF, McCarthy E, Eggan E, Bridge JA, Marcus SC. Association of Suicide Prevention Interventions With Subsequent Suicide Attempts, Linkage to Follow-up Care, and Depression Symptoms for Acute Care Settings: A Systematic Review and Meta-analysis. JAMA Psychiatry. 2020 Oct 1;77(10):1021-1030. doi: 10.1001/jamapsychiatry.2020.1586. |
| 20 | Study of individual-level interventions | Hofstra E, van Nieuwenhuizen C, Bakker M, Özgül D, Elfeddali I, de Jong SJ, van der Feltz-Cornelis CM. Effectiveness of suicide prevention interventions: A systematic review and meta-analysis. Gen Hosp Psychiatry. 2020 Mar-Apr;63:127-140. doi: 10.1016/j.genhosppsych.2019.04.011. |
| 21 | Not focussed on means restriction | White P, Skirrow H, George A, Memon A. A systematic review of economic evaluations of local authority commissioned preventative public health interventions in overweight and obesity, physical inactivity, alcohol and illicit drugs use and smoking cessation in the United Kingdom. J Public Health (Oxf). 2018 Dec 1;40(4):e521-e530. doi: 10.1093/pubmed/fdy026. |
| 22 | Study of individual-level interventions | Hawton K, Witt KG, Salisbury TLT, Arensman E, Gunnell D, Hazell P, Townsend E, van Heeringen K. Psychosocial interventions following self-harm in adults: a systematic review and meta-analysis. Lancet Psychiatry. 2016 Aug;3(8):740-750. doi: 10.1016/S2215-0366(16)30070-0. |
| 23 | study of individual-level interventions | Meerwijk EL, Parekh A, Oquendo MA, Allen IE, Franck LS, Lee KA. Direct versus indirect psychosocial and behavioural interventions to prevent suicide and suicide attempts: a systematic review and meta-analysis. Lancet Psychiatry. 2016 Jun;3(6):544-54. doi: 10.1016/S2215-0366(16)00064-X. |
| 24 | Not focussed on means restriction | Anglemyer A, Horvath T, Rutherford G. The accessibility of firearms and risk for suicide and homicide victimization among household members: a systematic review and meta-analysis. Ann Intern Med. 2014 Jan 21;160(2):101-10. doi: 10.7326/M13-1301. Erratum in: Ann Intern Med. 2014 May 6;160(9):658-9. |
| 25 | Not focussed on means restriction | Mishara BL, Côté LP, Dargis L. Systematic Review of Research and Interventions With Frequent Callers to Suicide Prevention Helplines and Crisis Centers. Crisis. 2023 Mar;44(2):154-167. doi: 10.1027/0227-5910/a000838. |
| 26 | Not focussed on means restriction | Grigore M. Havârneanu, Jean-Marie Burkhardt, Françoise Paran,  A systematic review of the literature on safety measures to prevent railway suicides and trespassing accidents, Accident Analysis & Prevention, Volume 81,  2015, Pages 30-50, https://doi.org/10.1016/j.aap.2015.04.012 |
| 27 | Not focussed on means restriction | Jeon SM, Lim H, Cheon HB, Ryu J, Kwon JW. Assessing the Labeling Information on Drugs Associated With Suicide Risk: Systematic Review. JMIR Public Health Surveill. 2024 Jan 30;10:e49755. doi: 10.2196/49755. |
| 28 | Not a systematic review | Altavini CS, Asciutti APR, Solis ACO, Wang YP. Revisiting evidence of primary prevention of suicide among adult populations: A systematic overview. J Affect Disord. 2022 Jan 15;297:641-656. doi: 10.1016/j.jad.2021.10.076. |
| 29 | Not a systematic review | van der Feltz-Cornelis CM, Sarchiapone M, Postuvan V, Volker D, Roskar S, Grum AT, Carli V, McDaid D, O'Connor R, Maxwell M, Ibelshäuser A, Van Audenhove C, Scheerder G, Sisask M, Gusmão R, Hegerl U. Best practice elements of multilevel suicide prevention strategies: a review of systematic reviews. Crisis. 2011;32(6):319-33. doi: 10.1027/0227-5910/a000109 |
| 30 | Clinical setting | Robinson J, Hetrick SE, Martin C. Preventing Suicide in Young People: Systematic Review. Australian & New Zealand Journal of Psychiatry. 2011;45(1):3-26. doi:10.3109/00048674.2010.511147 |
| 31 | Clinical setting | Gunnell D, Bennewith O, Hawton K, Simkin S, Kapur N. The epidemiology and prevention of suicide by hanging: a systematic review. Int J Epidemiol 2005; 34: 433–42 |
| 32 | Not focussed on means restriction | Jeon SM, Lim H, Cheon HB, Ryu J, Kwon JW. Assessing the Labeling Information on Drugs Associated With Suicide Risk: Systematic Review. JMIR Public Health Surveill. 2024 Jan 30;10:e49755. doi: 10.2196/49755. |
| 33 | not focussed on means restriction | Witt KG, Hetrick SE, Rajaram G, Hazell P, Taylor Salisbury TL, Townsend E, Hawton K. Interventions for self‐harm in children and adolescents. Cochrane Database of Systematic Reviews 2021, Issue 3. Art. No.: CD013667. DOI: 10.1002/14651858.CD013667.pub2. |
| 34 | Not a systematic review | Beautrais, A. (2007). Suicide by jumping: A review of research and prevention strategies. Crisis: The Journal of Crisis Intervention and Suicide Prevention, 28(Suppl 1), 58–63. https://doi.org/10.1027/0227-5910.28.S1.58 |
| 35 | Not a systematic review | Beautrais A, Fergusson D, Coggan C, Collings C, Doughty C, Ellis P, Hatcher S, Horwood J, Merry S, Mulder R, Poulton R, Surgenor L. Effective strategies for suicide prevention in New Zealand: a review of the evidence. N Z Med J. 2007 Mar 23;120(1251 |
| 36 | Not focussed on means restriction | Yip PS, Caine E, Yousuf S, Chang SS, Wu KC, Chen YY. Means restriction for suicide prevention. Lancet. 2012 Jun 23;379(9834):2393-9. doi: 10.1016/S0140-6736(12)60521-2. |
| 37 | Not focussed on means restriction | Ratnayake, R., Links, P. S., & Eynan, R. (2007). Suicidal behaviour on subway systems: A review of the epidemiology. Journal of Urban Health, 84, 766 |
| 38 | Not focussed on means restriction | Gunnell D, Eddleston M, Phillips MR, Konradsen F. The global distribution of fatal pesticide self-poisoning: systematic review. BMC Public Health. 2007 Dec 21;7:357. doi: 10.1186/1471-2458-7-357. |
| 39 | Not a systematic review | Ohsfeldt RL, Morrisey MA. Firearms, firearms injury, and gun control: a critical survey of the literature. Adv Health Econ Health Serv Res. 1992;13:65-82. |
| 40 | Not a systematic review | Daigle MS. Suicide prevention through means restriction: assessing the risk of substitution. A critical review and synthesis. Accid Anal Prev. 2005 Jul;37(4):625-32. doi: 10.1016/j.aap.2005.03.004. |
| 41 | Not a systematic review | Routley, V. (2007). Motor vehicle exhaust gas suicide: Review of countermeasures. Crisis: The Journal of Crisis Intervention and Suicide Prevention, 28(Suppl 1), 28–35. https://doi.org/10.1027/0227-5910.28.S1.28 |
| 42 | Not a systematic review | Matthew Miller, David Hemenway (1999) The relationship between firearms and suicide: A review of the literature, Aggression and Violent Behavior, Volume 4, Issue 1 |
| 43 | Not focussed on means restriction | Virginia Routley, Carolyn Staines, Chris Brennan, Narelle Haworth, Joan Ozanne-Smith (2003) Suicide and natural deaths in road traffic: review. |
| 44 | Not focussed on means restriction | Okolie 2017 Okolie C, Dennis M, Simon Thomas E, John A. A systematic review of interventions to prevent suicidal behaviors and reduce suicidal ideation in older people. Int Psychogeriatr. 2017 Nov;29(11):1801-1824. doi: 10.1017/S1041610217001430. |
| 45 | Not focussed on means restriction | Clifford AC, Doran CM, Tsey K. A systematic review of suicide prevention interventions targeting indigenous peoples in Australia, United States, Canada and New Zealand. BMC Public Health. 2013 May 13;13:463. doi: 10.1186/1471-2458-13-463. |

Table S4: Summary of included studies

| **Author and year** | **Category** | **Description of interventions included** | **Summary of study countries and income level** | **Study designs of included studies** | **Study quality/bias assessments** | **Study outcome measure/s** | **Main findings of the systematic review (including meta-analysis)** | **Risk estimate from meta-analysis** | **N events in non-intervention group** | **N events in intervention group** |
| --- | --- | --- | --- | --- | --- | --- | --- | --- | --- | --- |
| Okolie et al., 2020^19^ | Roads | No eligible studies | NA | NA | NA | NA | NA | NA | NA | NA |
| Okolie et al., 2020b ^16^ | Jumping | 14 studies of means restrictions at bridges and other jumping sites: Barriers on bridges or viaducts (6 studies), restricting road access to cliffs/headlands (2), safety net on building (1), guard rail on hospital building (1). Barriers were also studied in combination with signs for crisis helpline and measures to increase likelihood of human intervention (CCTV, patrols). One study compared several means restriction interventions at several jumping sites. | Switzerland (3) the USA (3), the UK (2), Canada (2), New Zealand (2), and Australia (2).  All high-income countries. | Before-and-after studies (13), cost-effectiveness analysis (1) | GRADE criteria: evidence assessed as low quality. Cochrane ROBINS-I tool: most studies/domains assessed as low, moderate or unclear risk of bias. | Number of suicide deaths (13 studies), suicide attempts (1)  Included analysis of unintended consequences. | Meta-analysis of 12 studies showed IRR for suicide in favour of the intervention.  Conclusions were based on low quality evidence largely due to the observational nature of the studies. | IRR 0.09 (0.03 to 0.27), P < 0.001; I2 = 88.40%  9 studies of jumping interventions delivered in isolation:  IRR = 0.05, 95% CI  0.01 to 0.16 | 742.3 suicide deaths per year | 70.6 suicide deaths per year |
| Pirkis et al., 2013^2^ | Jumping | 9 studies of 8 structural interventions on bridges and cliffs | UK (2), USA (2), New Zealand (2), Canada (1), Switzerland (1). All high-income countries. | Observational before-and-after/interrupted time series. | None recorded. | Numbers and rates of suicide deaths.  5 included details on the height of barrier.  Included analysis of unintended consequences. | 86% (95% CI 79% to 91%) reduction in jumping suicides per year at the intervention sites.  44% (95% CI 15% to 81%) increase in jumping suicides per year at nearby sites.  Net gain was 28% (95% CI 13% to 40%) reduction in all jumping suicides per year in the study cities. | RR 0.14, 95% CI 0.09 to 0.21, P = 0.001 | 436 (5.7 per year) | 21 (0.5 per year) |
| Barker et al., 2017^20^ | Railway | 5 articles of means restriction measures at railway stations including platform screen doors (3 studies). | Hong Kong (2), Japan (1).  All high-income countries. | Observational before-and-after design.  Outcomes were measured at control locations in 4 studies. | No formal assessments conducted. | Number of suicide deaths (6), fatality rate of suicide incidents (2), suicide attempts (2).  Included analysis of unintended consequences. | ‘Strong’ evidence for the effectiveness of platform screen doors for reducing suicides. | Meta-analysis was not conducted. | Not available | Not available |
| Lim et al., 2021^21^ | Poisoning | 62 studies of means restriction of pesticides (23 studies), domestic gas (17), motor vehicle exhaust (11) and pharmaceuticals (11). | 26 countries were represented in the studies.  20 high-income countries (53 studies)  6 low- and middle-income countries (LMIC) (9 studies). | Studies reporting changes in suicide rates before and after the intervention. | ROBINS-I tool for uncontrolled before and after studies.  Most studies had at least some low or medium risk of bias. Risk of confounding bias was highest in studies with limited trend analysis/preintervention data. | Change in rate of suicide deaths using the restricted poison, measured using IRR.  Included analysis of unintended consequences. | Evidence of reduced incidence of suicide from pesticide poisoning in 6/7 studies eligible for calculating IRR estimates.  Reduced incidence of suicide by domestic gas in all studies in which IRR could be calculated, with some increases in suicide by other means.  Evidence of reduced suicides by motor exhaust in 4/8 studies eligible for IRR calculation.  Pharmaceuticals: reduction in suicides by poisoning in 6/7 eligible studies. | Meta-analysis was not conducted.  Standardised IRRs were calculated for each primary study.  The median change in method-specific suicide rates was −1.18 (−2.03 to −0.46) per 100 000  people. | Not available | Not available |
| Morgan et al., 2005^22^ | Paracetamol poisoning | 12 studies examining the 1998 restrictions of paracetamol in the UK. | All 12 studies were of the UK | Observational before-and-after studies. | No formal assessments conducted but study design features of the included studies were discussed briefly. | Severity of paracetamol poisoning (8 studies), hospital admission (6) admissions  to liver transplant units (3), deaths from paracetamol poisoning (3) and sales of paracetamol (2).  Some studies measured more than one outcome. | Poisoning severity: 3 out of 8 studies reported reductions;  Hospital admissions: 5 out of 6 reported reduced number of paracetamol poisonings; Admissions to liver transplant units: all 3 reported reductions in admissions and transplants; Mortality: 1 out of 3 studies reported reductions in mortality; Over-the-counter sales: both studies reported reductions. | Meta-analysis was not conducted. | Not available | Not available |
| Gunnell et al., 2017^4^ | Pesticides | 27 studies of national bans of specific pesticides (12 studies), pesticide sales restrictions (8) and mandatory licensing or registering of users (5). | Studies included 16 countries. 5 low-income or middle-income countries  (Bangladesh, Colombia, India, Jordan and Sri Lanka), and 11 high-income countries (Denmark, Finland, Germany,  Greece, Hungary, Ireland, Japan, South Korea, Taiwan, UK, and USA). | Before-and-after observational studies, some including time trend analysis or geographical control areas. | Risk of bias assessments conducted using Cochrane Effective Practice and Organisation of Care assessment criteria for interrupted time series studies.  Few studies accounted for pre-legislation trends in suicide rates or other factors associated with changes in suicide rates. | Rates and numbers of pesticide suicide deaths.  Included analysis of unintended consequences. | Narrative synthesis.  National bans for specific pesticides were followed by  reductions in pesticide suicides and, in 3/6 countries, falls in overall suicide mortality.  Four of the seven studies of sales restrictions  showed sales restrictions were followed by decreases in pesticide suicides, but studies were lower quality.  One of the  two studies investigating trends in overall suicide mortality reported a fall in deaths, but there were also  decreases in suicide deaths from other methods. | Meta-analysis was not conducted. | Not available | Not available |
| Reifels et al., 2019^23^ | Pesticides | 5 studies of non-pesticide-based pest management(NPM) (1), central, lockable  pesticide storage boxes (1), household-basedlockable pesticide storage containers (1) and bans on specific pesticides (2). | 3 studies in India and 2 in Sri Lanka (all LMICs) | 2 cluster randomized  controlled trials, 2  quasi-experimental designs and one before-and-after design. | Study quality is discussed in depth but no formal quality assessment presented. | Rates of suicide (4), suicide from pesticide poisoning (2) and attempted suicide (3).  Included analysis of unintended consequences. | One randomised study found no evidence of effectiveness of household-based lockable storage.  Four studies found limited evidence for effectiveness of other interventions, though there were methodological limitations. | Meta-analysis was not conducted. | Not available | Not available |
| Pirkis et al., 2015^17^ | Jumping | 23 articles representing 18 unique studies. Multiple means restriction interventions (jumping from height = 15, carbon monoxide poisoning = 2 and jumping in front of moving object = 1).  Of the 15 jumping from height interventions, information on height of barrier was included in 9.  Other interventions were also included: encouraging help-seeking and increasing the likelihood of intervention by a third party. | All high-income countries: USA (5), UK (3),  NZ (2),  Australia (2),  Canada (2),  Hong Kong (2),  Switzerland (2). | Studies reporting changes in suicide rates before and after the intervention. | None recorded | Suicide rates | Interventions that  restricted access to means were associated with a reduction in the number of suicides per year. | Pooled IRR for 11 studies considering the intervention in isolation  (IRR 0·07, 95% CI 0·02–0·19, p<0·0001). | 863 (5.8 per year) | 211 (2.4 per year) |
| Cox et al., 2013^24^ | Jumping | 19 articles describing 14 studies. Nine studies of restricting  access to means by installing physical barriers at ‘hotspot’ sites used for jumping from a height or jumping in front of a train.  The review also included studies of non-means restriction interventions including encouraging help-seeking, increasing the likelihood of intervention by a third party and encouraging responsible media reporting of suicide at hotspot sites. | UK (2), Hong Kong (1), New Zealand (2), US (2), Switzerland (1), Canada (1).  All high-income countries. | Observational before-and-after/interrupted time series.  One study included quasi-experimental design element using comparison railway stations without platform screen doors. | No formal assessments conducted but study design features of the included studies were discussed. | Numbers of suicide deaths.  Included analysis of unintended consequences. | Reduction to zero suicides in 3 studies of barriers and 1 of restricting road access.  Decreases in suicides in 4 studies on bridge barriers and 1 study of platform screen doors.  Substantial overlap in included studies with Pirkis et al., 2013. | Meta-analysis was not conducted. | Not available | Not available |
| Linskens et al., 2023^25^ | Multiple:  Jumping;  Domestic gas, motor vehicle gas and charcoal poisoning | 10 studies of 9 interventions including 4 studies of barriers at bridges or railway stations, 3 of platform screen doors sand 3 of restricting access to charcoal.  The review also included studies of non-means restriction interventions including housing, workplace initiatives, awareness campaigns, screening, gatekeeper training and school-based programmes. | Hong Kong (2), South Korea (2), Taiwan, Canada, Australia, Switzerland, Japan.  All high-income countries. | All observational studies, 6 with concurrent control group and 4 pre-post designs. | Cochrane Risk of Bias 1.0 instrument to  assess the quality of RCTs and cluster RCTs and a modified version of the Joanna Briggs Institute Critical Appraisal Tool for Quasi-Experimental Studies to  assess the quality of observational studies.  GRADE framework was used to rate the certainty of evidence. | Suicide deaths (including method-specific) per year. | Three studies found (with low certainty) that restricting access to charcoal may reduce suicides by charcoal poisoning. Seven studies found (with low certainty) that installing barriers may reduce suicide deaths at bridges and railway stations. | Meta-analysis was not conducted. | Not available | Not available |
| Ishimo et al., 2021^26^ | Multiple:  Jumping; Pesticides;  Domestic gas, motor vehicle gas and charcoal poisoning | 11 studies on physical barriers (railway and viaduct barriers = 7, platform screen doors = 2, a safety net and restricting access to a cliff). 3 studies on pesticides restrictions. 4 studies on domestic gas, motor vehicle gas and charcoal poisoning. | 3 studies (of 2 interventions) in Canada, Japan (3 studies), New Zealand (2), Switzerland (2), South Korea (1), UK (1) Australia (1).  All high-income countries. | Time series (7), pre-post with no control group (4) quasi-experimental, with control group at non-intervention sites (2). | Study quality assessed using the Effective Public Health Practice Project (EPHPP) Quality Assessment  Tool. The evidence was assessed as moderate to strong. | Suicide mortality rates and site/method-specific suicide deaths.  Included analysis of unintended consequences. | Eleven studies found statistically significant associations with  physical barriers and reduced suicide mortality.  Two studies found both null effects and statistically  significant associations with reducing suicide mortality.  Only 1 study (of a. bridge barrier) reported results by sex, finding evidence for reduction in suicide for men but not women. | Meta-analysis was not conducted. | Not available | Not available |
| Zalsman et al., 2016^27^ | Multiple:  Jumping; Pesticides; Domestic gas, motor vehicle gas and charcoal poisoning; Firearms; Medication poisoning | 24 studies of firearms restrictions (11), medication withdrawal (5), pesticide restriction (5), barriers at jumping sites (2) and charcoal sales restrictions (1).  The review also included studies on public and physician education, media strategies, screening, treatments, and internet or hotline support.  The review also included 5 systematic reviews: 2 met our inclusion criteria so were included in our umbrella review, 3 did not meet our inclusion criteria (2 were of clinical populations and 1 was not a review of means restriction). | USA (4), Australia (2), UK (3), Sri Lanka (3), India (1), Norway (1), Sweden (1), Denmark (1), Switzerland (1), Israel (1), New Zealand (1), Austria (1), Canada (1), Hong Kong (1).  4/24 studies in LMICs. | 5 ecological studies,  5 quasi-experimental studies (including 4 pre-post and 1 interrupted time series designs), one case-control study. | Studies were rated by 18 suicide prevention experts using the Oxford Centre For Evidence Based Medicine criteria.  The majority on included means restrictions studies were rated 2c (evidence from ecological studies).  However, study quality was not assessed. | Suicide death, including methods-specific death.  Included analysis of unintended consequences. | Some evidence showed firearms restrictions were associated with reduced suicides, though evidence from other studies was mixed.  Evidence was stronger for the effect of reducing analgesic pack sizes on suicide deaths.  Restrictions on pesticide availability contributed to reduced suicides in countries where it was a commonly used method.  There was strong evidence for barriers at sites used for jumping, for detoxification of domestic gas and the introduction of catalytic converters in cars. | Meta-analysis was not conducted. | Not available | Not available |
| Mann et al., 2005^28^ | Multiple:  Jumping; Pesticides; Domestic gas, motor vehicle gas and charcoal poisoning; Firearms; Medication poisoning | 27 studies: 7 studies of barbiturate restrictions, 6 of firearms restrictions, 6 of domestic gas detoxification, 4 on catalytic converters, 2 of pesticide restrictions, 1 on barriers to jumping, 1 on analgesic pack size change.  The review also included studies on clinical interventions, screening, physician education and medication. | US (5), Canada (3), Australia (4), Finland (1), Samoa (1), UK (5), Japan (1), New Zealand (1), Germany (1), Norway (1), Sweden (1), Netherlands (1), Denmark (1). | One quasi-experimental (cohort design), 27 ecological designs. | Level of evidence was rated according to the Oxford Centre For Evidence Based Medicine criteria.  All the ecological studies were identified as having a 2c rating.  However, study quality was not assessed. | Suicide deaths.  Included analysis of unintended consequences. | Where the method was common, suicides decreased after restrictions on firearms, pesticides, barbiturates and analgesics, detoxification of domestic gas, the introduction of mandatory catalytic converters in cars and barriers at jumping sites.  Confounding factors such as concurrent increases in antidepressant use should also be considered when interpreting findings. | Meta-analysis was not conducted. | Not available | Not available |
| Mann et al., 2021^29^ | Multiple:  Pesticides; Domestic gas, motor vehicle gas and charcoal poisoning; Firearms | 49 studies of firearms restrictions.  2 studies of pesticides, 3 studies of domestic and motor vehicle gas. The list of included means restriction studies is missing from the online supplement. | List of included studies not available. | List of included studies not available. | No formal assessment of study quality. | Suicide deaths. | Pesticides restrictions contributed to reduction in suicides.  Firearms restrictions was associated with reduced suicide with some method substitution.  Catalytic converters, domestic gas detoxification and barriers at jumping sites are ‘proven’ approaches. | Meta-analysis was not conducted. | Not available | Not available |
| Robinson et al 2018^30^ | Multiple:  Pesticides; Firearms; Medication poisoning | 8 studies on means restriction (2 on restriction of SSRIs in under 18s, 6 on firearms restrictions).  Included interventions that targeted or included data on young people (aged between 12 and 25 years or mean age between 12 and 25 years). | Canada (3), New Zealand (1), Israel (1), Austria (1), UK (1).  Plus one study of 23 WHO Stratum A countries. | Interrupted time series, 2 with a control group. | Studies were assessed for whether they included data from multiple time points pre and post intervention and whether data collection was affected by the intervention.  5/6 studies on firearms and both studies in SSRIs included multiple time points.  All interventions were assessed as unlikely to impact data collection. | Suicide deaths, including method-specific.  Included analysis of unintended consequences. | Five out of six studies of firearms restrictions reported decreases in youth suicide deaths.  Two studies on SSRI restrictions found no association with suicide rates. | Meta-analysis not conducted (only conducted for RCTs). | Not available | Not available |
| Bailey et al., 2023^31^ | Pesticide poisoning | Only one study on means restriction (pesticides) was included.  This was an update to Robinson et al., 2018.  Included interventions that targeted or included data on young people (mean age between 12 and 25 years). | Sri Lanka (1) | Cluster randomised controlled trial. | Risk of bias was assessed using the Cochrane risk of bias tool.  The included study was assessed as having low risk of bias. | Pesticide self-poisoning. | The findings for young people were not adequately powered to detect differences. | Meta-analysis was not conducted. | Not available | Not available |
| Hahn et al., 2005^32^ | Firearms | 15 studies of firearms restrictions.    The review included multiple violence outcomes with 15 focussed on suicide outcomes. | US (14) and Canada (2)*.  *One study included both US and Canada. | Time series with concurrent comparison group (4),  cross-sectional (11). | Study quality was assessed using the Community Guide methods, which includes quality of describing study PICO and methods to address confounding and other bias. | Suicide deaths.  Included analysis of unintended consequences. | Insufficient evidence was found to conclude whether firearms restrictions affect suicide rates, largely due to limitations in study designs. | Meta-analysis was not conducted. | Not available | Not available |
| National Institute for Health and Care Excellence, 2018^18^ | Multiple: Jumping; Firearms | 16 studies of means restriction including barriers and safety nets at jumping sites (11), platform screen doors (2), road access restriction (2) and firearm legislation (1).  The review also included studies on blue LED lamps (2), encouraging help-seeking (4) and surveillance (3). | Canada (2), US (3), New Zealand (2), Switzerland (1), UK (2), Austria (1), Australia (3), South Korea (1), Japan (1). | Before-and-after (14), retrospective observational (1), interrupted time series (1). | Evidence assessed using GRADE as moderate (safety nets, platform screen doors, road access restriction)) to high (barriers at jumping sites). | Suicide deaths and suicide attempts.  Included analysis of unintended consequences. | A substantial overall positive effect on suicide prevention for physical barriers at sites used for jumping. Further research is needed for platform screen doors.  A non-significant increase in suicides at other sites was found following physical barriers at jumping sites, with moderate certainty in the evidence. | A meta-analysis of 11 studies found a reduction in the number of suicides at sites where physical barriers were installed (risk ratio = 0.24 [95% confidence interval, 0.14 to 0.39]). | 1001 (3.16 per year) | 116 (0.72 per year) |
| Rubbo et al., 2025^33^ | Pesticides | 9 studies of pesticide regulations comprising nationwide or regional bans on specific substances. | India (2), South Korea (2), Taiwan (2), Japan (1), China (1), Mongolia (1).  4/9 studies were in LMICs. | Time series analyses (6), linear regression methods (3).  6/9 studies took into account secular trends. | Risks of bias in primary studies were assessed using modified risk of bias criteria for interrupted time series studies. One study had a low risk of bias in all domains and 8 had high or unclear risk of bias in one or more domains. | Suicides by pesticide poisoning.  In a post-hoc analysis, authors obtained raw data to estimate percentage change in rates of pesticide suicide and overall suicide. | All 9 studies showed reductions in pesticide suicide rates following bans, ranging from 28% to 92%. Four out of 5 studies reported decreases in suicide rates overall (range 7.0% to 45.1%). | Meta-analysis was not conducted. | Not available | Not available |


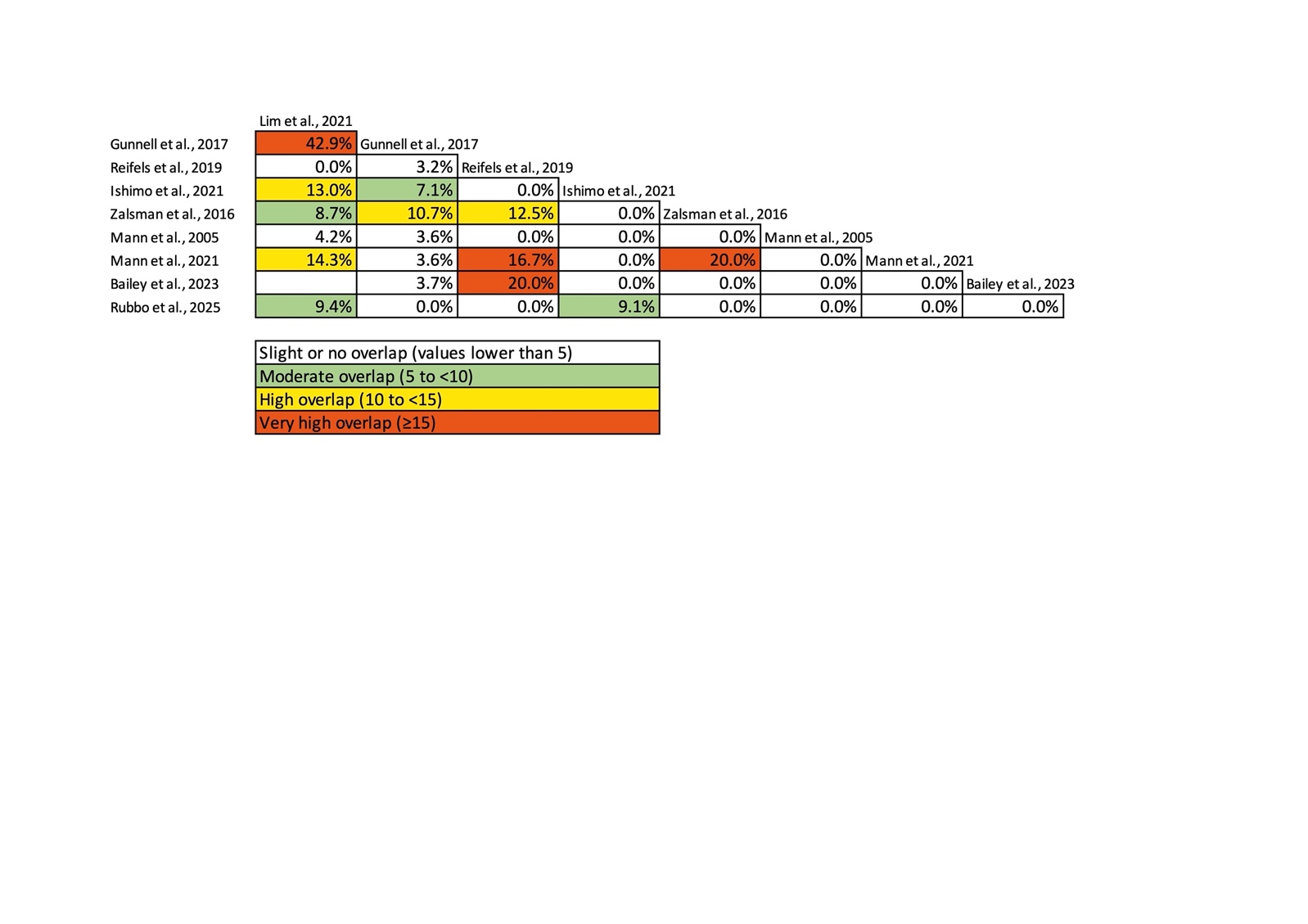


S1 Heat map


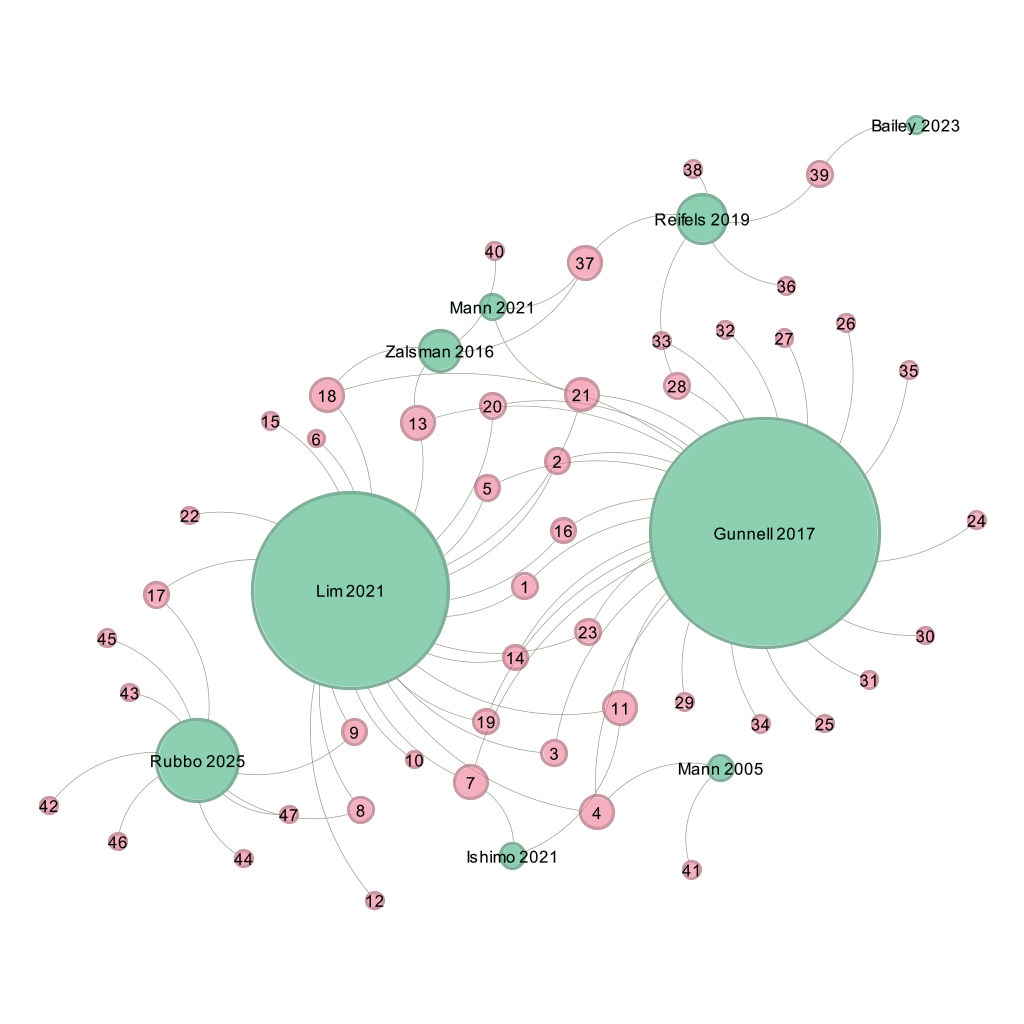


S2 Network diagram^1^

***Figure S1 and S2****: Heat map for primary study overlap analysis between systematic review pairs (S1) and network diagram (S2) for pesticide means restriction interventions (N = 9 reviews)*

^1^ Green nodes represent systematic reviews, pink nodes represent primary studies. Larger nodes denote higher numbers of edges. See Table S4 for primary study ID number and corresponding reference.


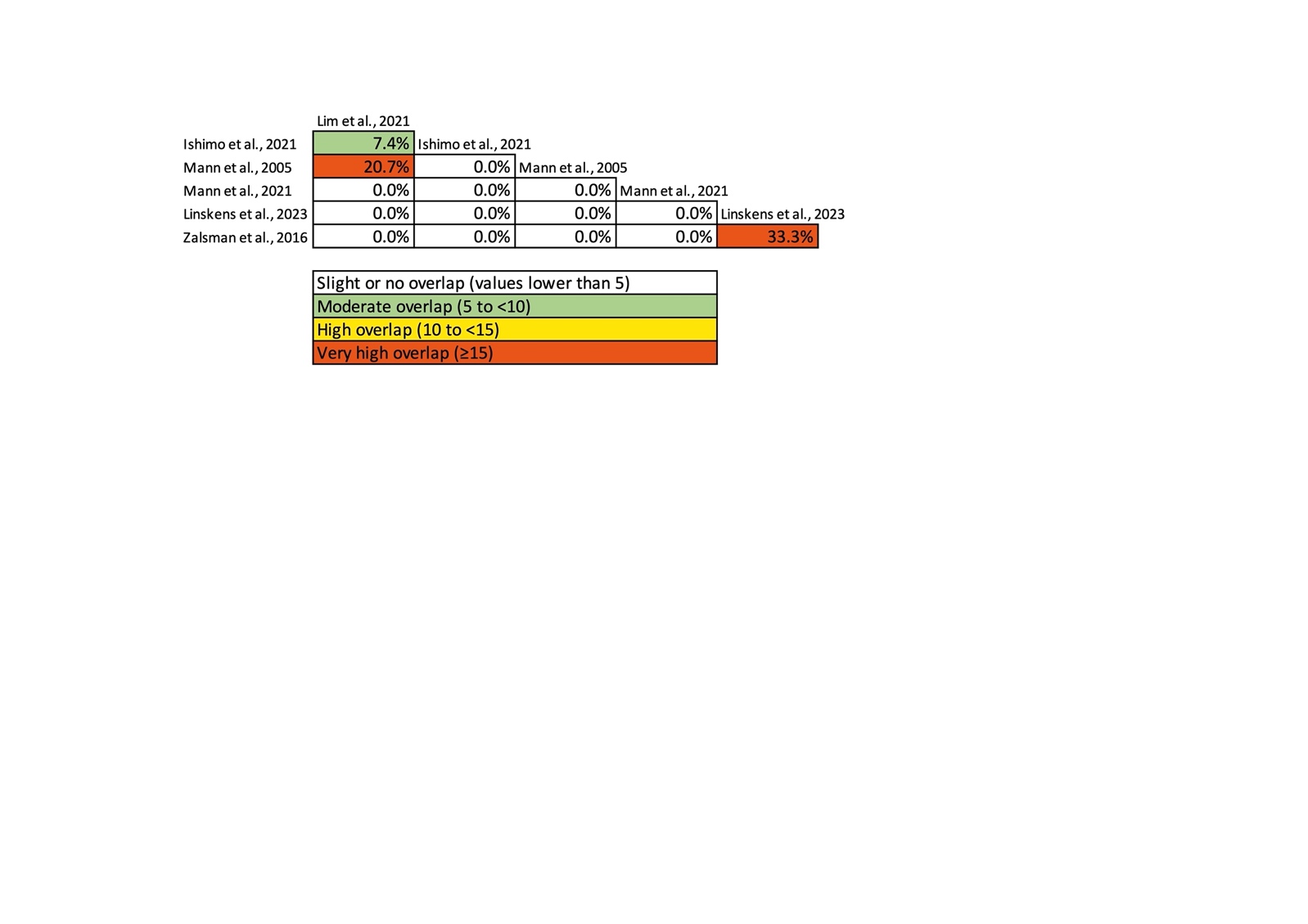


(S3) Heat map

**
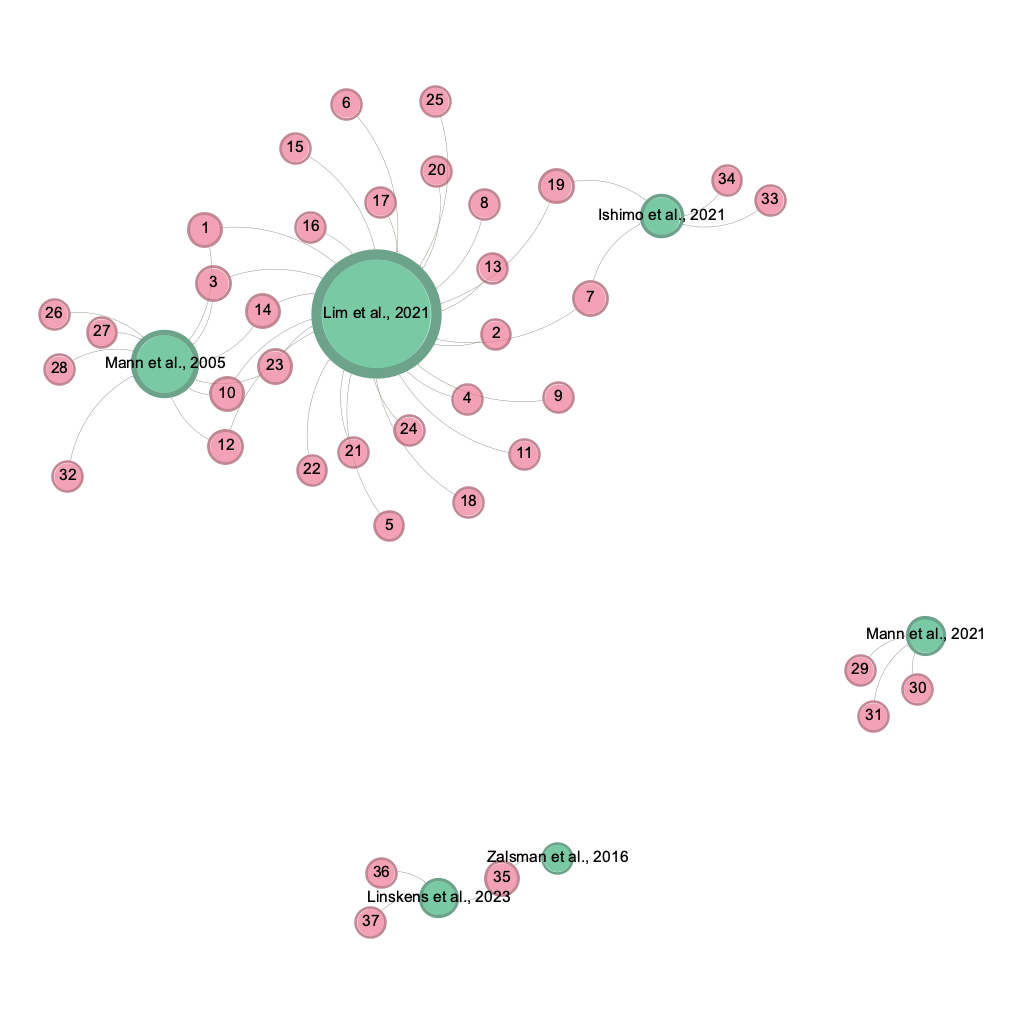
**

(S4) Network diagram

***Figure S3 and S4****: Heat map for primary study overlap analysis between systematic review pairs (S3) and network diagram (S4) for domestic gas, motor vehicle gas and charcoal restriction interventions (N = 6 reviews)*


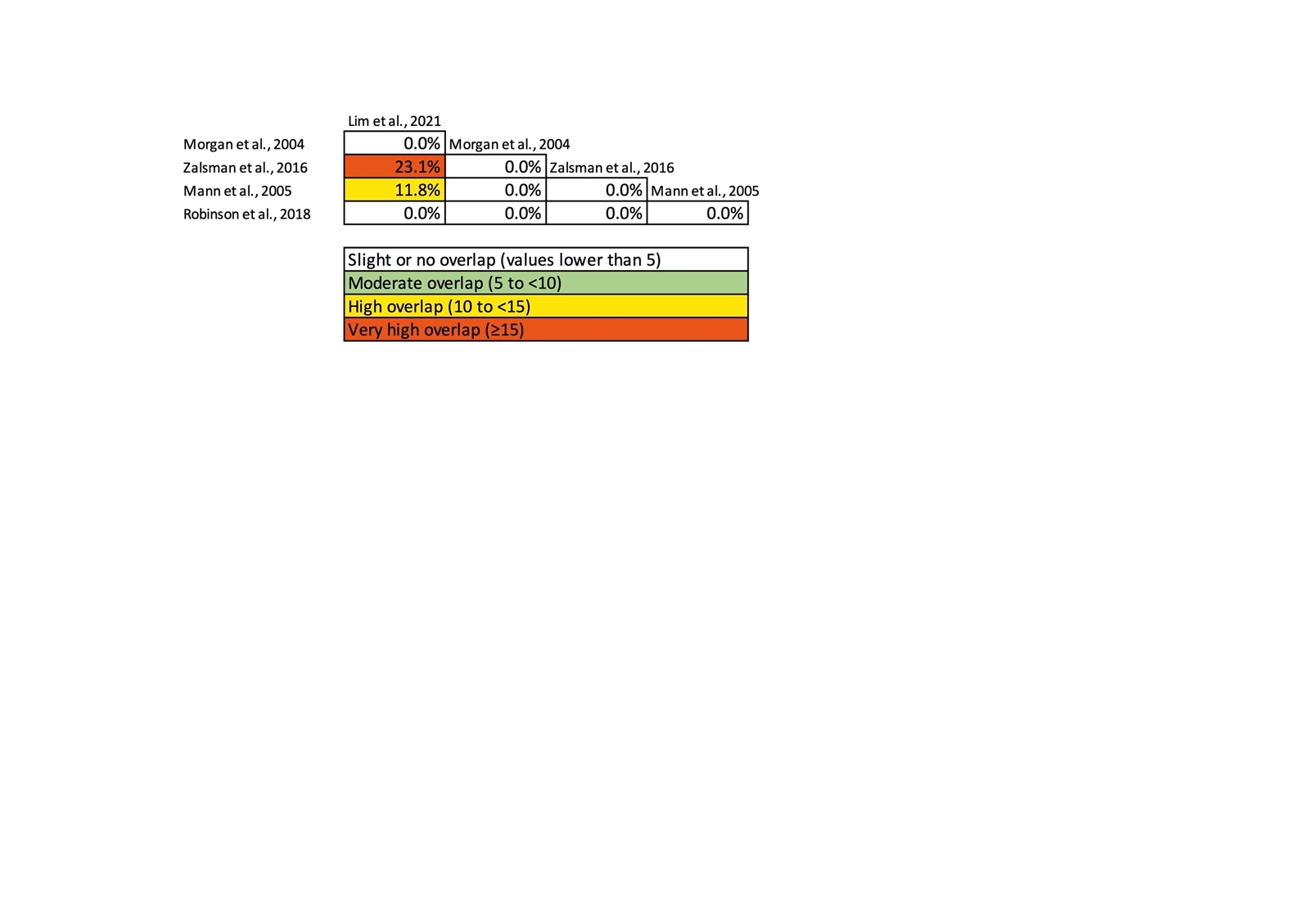


(S5) Heat map


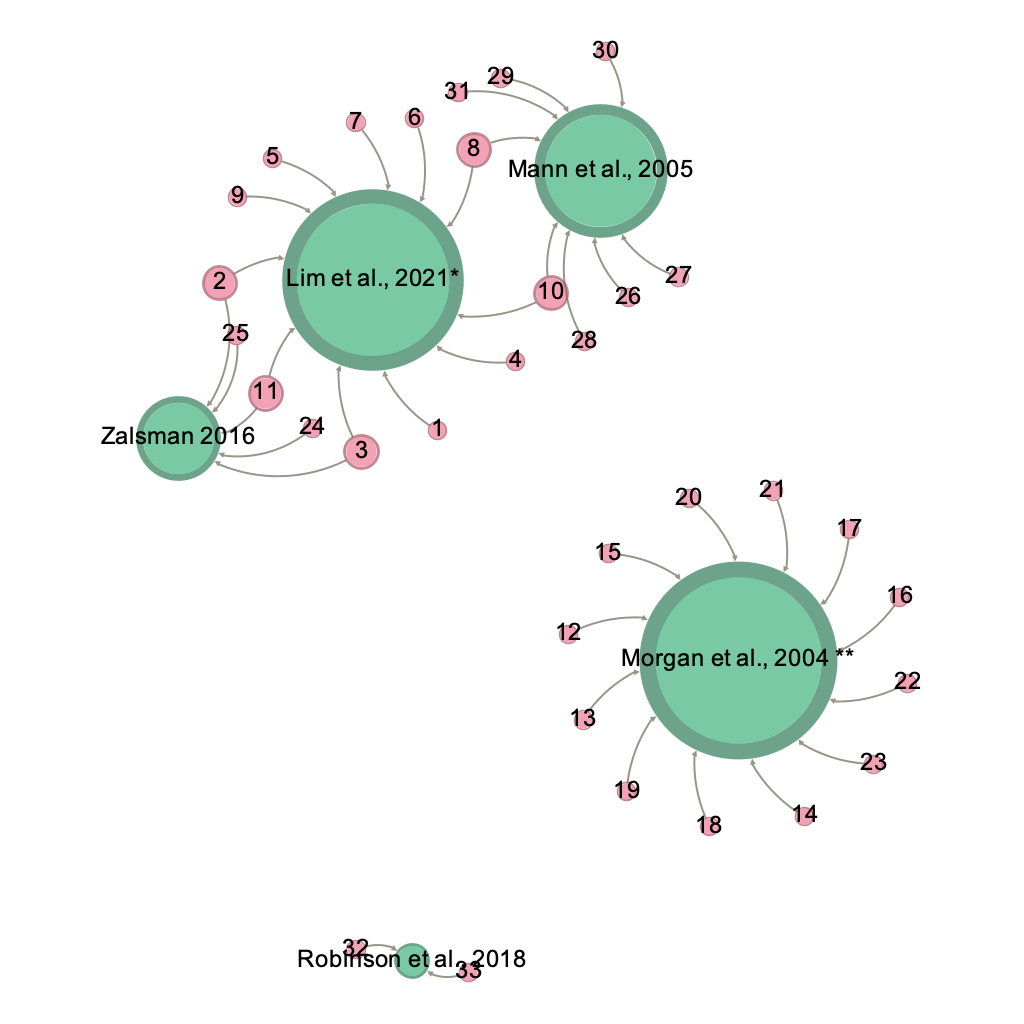


(S6) Network diagram

***Figure S5 and S6****: Heat map for primary study overlap analysis between systematic review pairs (S5) and network diagram (S6) for medication restriction interventions (N = 5 reviews)*


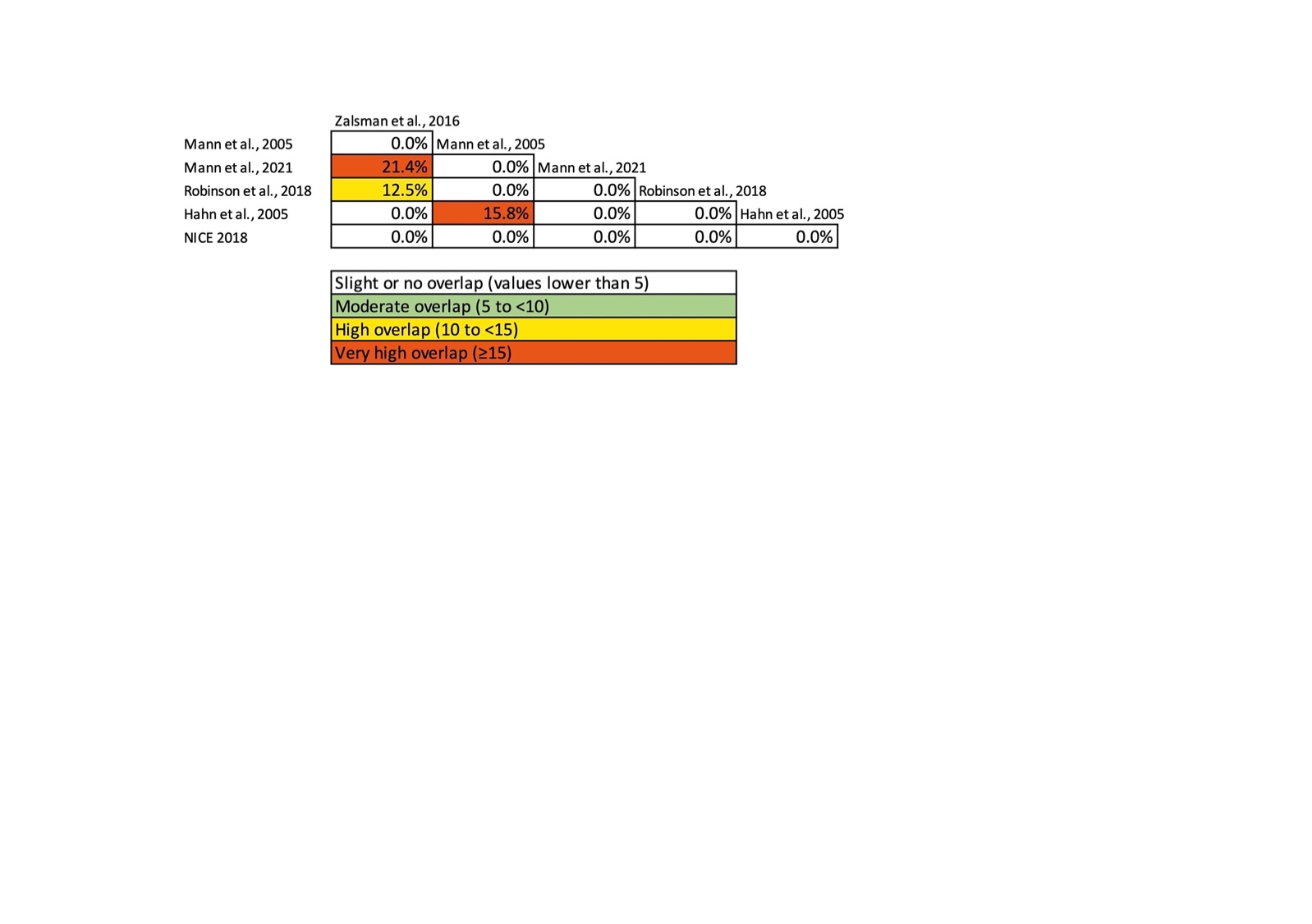


(S7) Heat map


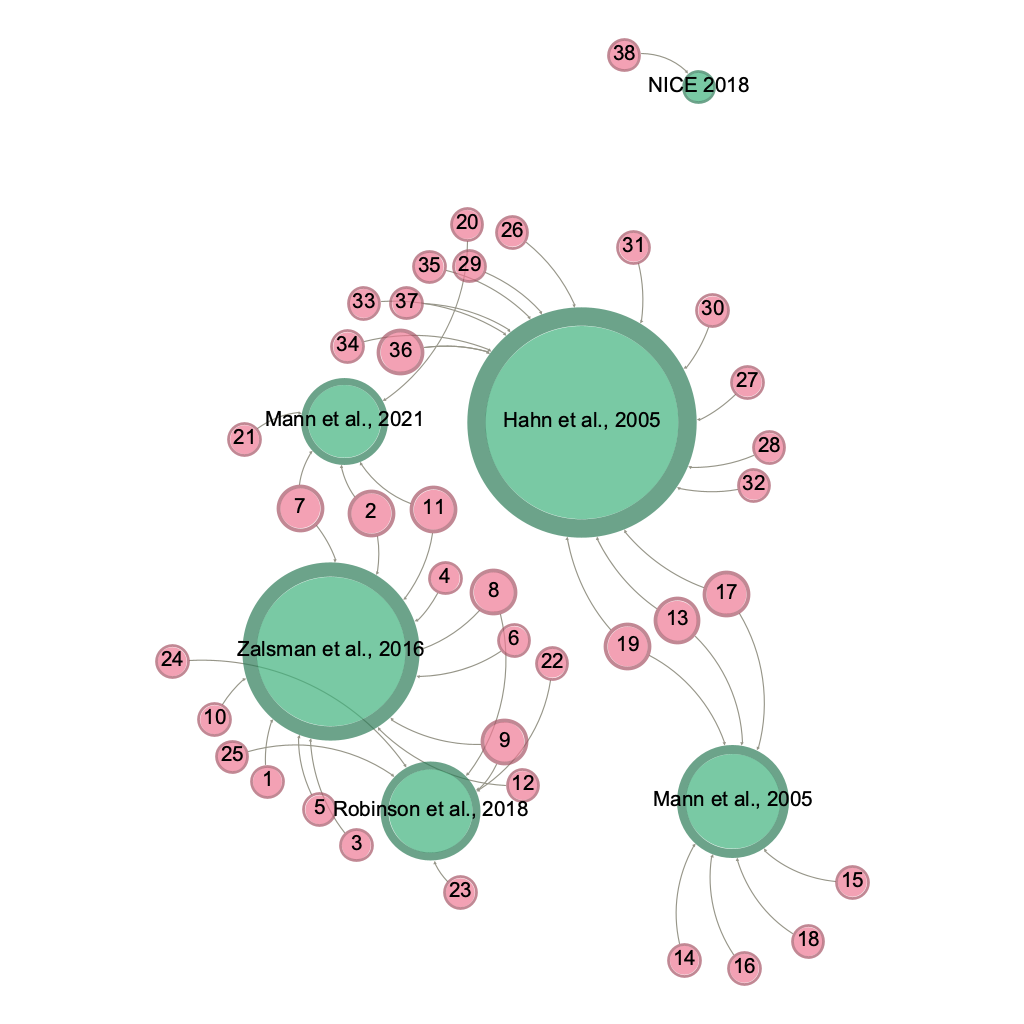


(S8) Network diagram

***Figure S7 and S8****: Heat map for primary study overlap analysis between systematic review pairs (S7) and network diagram (S8) for firearm interventions (N = 6 reviews)*

Table S5: Node labels for Figures 3, S2, S4, S8 and S8 and corresponding references

| **Figure 3 node label** | **Corresponding reference** |
| --- | --- |
| 1 | Beautrais A, Gibb S, Fergusson D, et al. Removing bridge barriers stimulates suicides: an unfortunate natural experiment. *AUSTRALIAN AND NEW ZEALAND JOURNAL OF PSYCHIATRY* 2009;43(6):495-97. doi: 10.1080/00048670902873714 |
| 2 | Bennewith O, Nowers M, Gunnell D. Effect of barriers on the Clifton suspension bridge, England, on local patterns of suicide: implications for prevention. *BRITISH JOURNAL OF PSYCHIATRY* 2007;190:266-67. doi: 10.1192/bjp.bp.106.027136 |
| 3 | Hemmer A, Meier P, Reisch T. Comparing Different Suicide Prevention Measures at Bridges and Buildings: Lessons We Have Learned from a National Survey in Switzerland. *PLOS ONE* 2017;12(1) doi: 10.1371/journal.pone.0169625 |
| 4 | Isaac M, Bennett J. Prevention of suicide by jumping: The impact of restriction of access at Beachy Head, Sussex during the foot and mouth crisis 2001. *Public Health Medicine* 2005; 6. |
| 5 | Law C, Sveticic J, De Leo D. Restricting access to a suicide hotspot does not shift the problem to another location. An experiment of two river bridges in Brisbane, Australia. *AUSTRALIAN AND NEW ZEALAND JOURNAL OF PUBLIC HEALTH* 2014;38(2):134-38. doi: 10.1111/1753-6405.12157 |
| 6 | \| Lester, D. Suicide from bridges in Washington DC. *PERCEPTUAL AND MOTOR SKILLS* 1993; 77: 534-534 doi: 10.2466/pms.1993.77.2.534 \| \| --- \| |
| 7 | Lockley A, Cheung Y, Cox G, et al. Preventing Suicide at Suicide Hotspots: A Case Study from Australia. SUICIDE AND LIFE-THREATENING BEHAVIOR 2014;44(4):392-407. doi: 10.1111/sltb.12080 |
| 8 | Mohl A, Stulz N, Martin A, et al. The "Suicide Guard Rail": a minimal structural intervention in hospitals reduces suicide jumps. *BMC research notes* 2012;5:408. doi: 10.1186/1756-0500-5-408 |
| 9 | Pelletier A. Preventing suicide by jumping: the effect of a bridge safety fence. *INJURY PREVENTION* 2007;13(1):57-59. doi: 10.1136/ip.2006.013748 |
| 10 | Perron S, Burrows S, Fournier M, et al. Installation of a Bridge Barrier as a Suicide Prevention Strategy in Montreal, Quebec, Canada. *AMERICAN JOURNAL OF PUBLIC HEALTH* 2013;103(7):1235-39. doi: 10.2105/AJPH.2012.301089 |
| 11 | Reisch T, Michel K. Securing a suicide hot spot: Effects of a safety net at the Bern Muenster Terrace. *SUICIDE AND LIFE-THREATENING BEHAVIOR* 2005;35(4):460-67. doi: 10.1521/suli.2005.35.4.460 |
| 12 | Sinyor M, Levitt A. Effect of a barrier at Bloor Street Viaduct on suicide rates in Toronto: natural experiment. *BRITISH MEDICAL JOURNAL* 2010;341 doi: 10.1136/bmj.c2884 |
| 13 | Skegg K, Herbison P. Effect of restricting access to a suicide jumping site. *AUSTRALIAN AND NEW ZEALAND JOURNAL OF PSYCHIATRY* 2009;43(6):498-502. doi: 10.1080/00048670902873698 |
| 14 | Whitmer D, Woods D. Analysis of the Cost Effectiveness of a Suicide Barrier on the Golden Gate Bridge. *CRISIS-THE JOURNAL OF CRISIS INTERVENTION AND SUICIDE PREVENTION* 2013;34(2):98-106. doi: 10.1027/0227-5910/a000179 |
| 15 | Beautrais A. Effectiveness of barriers at suicide jumping sites: a case study. *AUSTRALIAN AND NEW ZEALAND JOURNAL OF PSYCHIATRY* 2001;35(5):557-62. doi: 10.1046/j.1440-1614.2001.00951.x |
| 16 | Bennewith O, Nowers M, Gunnell D. Suicidal behaviour and suicide from the Clifton Suspension Bridge, Bristol and surrounding area in the UK: 1994-2003. *EUROPEAN JOURNAL OF PUBLIC HEALTH* 2011;21(2):204-08. doi: 10.1093/eurpub/ckq092 |
| 17 | Law C, Yip P, Chan W, et al. Evaluating the effectiveness of barrier installation for preventing railway suicides in Hong Kong. *JOURNAL OF AFFECTIVE DISORDERS* 2009;114(1-3):254-62. doi: 10.1016/j.jad.2008.07.021 |
| 18 | Law C, Yip P. An economic evaluation of setting up physical barriers in railway stations for preventing railway injury: evidence from Hong Kong. *JOURNAL OF EPIDEMIOLOGY AND COMMUNITY HEALTH* 2011;65(10):915-20. doi: 10.1136/jech.2010.115188 |
| 19 | O’CARROLL P, SILVERMAN M, BERMAN A. COMMUNITY SUICIDE-PREVENTION - THE EFFECTIVENESS OF BRIDGE BARRIERS. *SUICIDE AND LIFE-THREATENING BEHAVIOR* 1994;24(1):89-99. |
| 20 | Sinyor M, Schaffer A, Redelmeier DA, et al. Did the suicide barrier work after all? Revisiting the Bloor Viaduct natural experiment and its impact on suicide rates in Toronto. BMJ Open 2017;**7**:e015299. doi:10.1136/ bmjopen-2016-015299 |
| 21 | Ueda M, Sawada Y, Matsubayashi T. The effectiveness of installing physical barriers for preventing railway suicides and accidents: Evidence from Japan. *JOURNAL OF AFFECTIVE DISORDERS* 2015;178:1-4. doi: 10.1016/j.jad.2015.02.017 |
| 22 | Chung Y, Kang S, Matsubayashi T, et al. The effectiveness of platform screen doors for the prevention of subway suicides in South Korea. *JOURNAL OF AFFECTIVE DISORDERS* 2016;194:80-83. doi: 10.1016/j.jad.2016.01.026 |

| **Figure S2 node label** | **Corresponding reference** |
| --- | --- |
| 1 | ONYON L, VOLANS G. THE EPIDEMIOLOGY AND PREVENTION OF PARAQUAT POISONING. *HUMAN TOXICOLOGY* 1987;6(1):19-29. doi: 10.1177/096032718700600104 |
| 2 | Fitzgerald GR, Barniville G, Flanagan M, Silke B, Carmody M, O’DwyerWF. The changing pattern of paraquat poisoning: an epidemiologic study. Ir Med J. 1978;71(4):103-108. |
| 3 | Moebus S, B.dekerW. Mortality of intentional and unintentional pesticide poisonings in Germany from 1980 to 2010. J Public Health Policy. 2015;36(2):170-180. doi:10.1057/jphp.2014.56 |
| 4 | Ohberg A, Lonnqvist J, Sarna S, Vuori E, Penttila A. Trends and availability of suicide methods in Finland: proposals for restrictive measures. Br J Psychiatry. 1995;166(1):35-43. doi:10.1192/bjp.166.1.35 |
| 5 | Kastanaki AE, Kraniotis CF, Kranioti EF, Nathena D, Theodorakis PN, Michalodimitrakis M. Suicide by pesticide poisoning: findings from the island of Crete, Greece. Crisis. 2010;31(6):328-334. doi:10.1027/0227-5910/a000042 |
| 6 | Kerv.gant M, Merigot L, Glaizal M, Schmitt C, Tichadou L, de Haro L. Paraquat poisonings in France during the European ban: experience of the Poison Control Center in Marseille. J Med Toxicol. 2013;9(2):144-147. doi:10.1007/s13181-012-0283-6 |
| 7 | Cha ES, Chang SS, Gunnell D, Eddleston M, Khang YH, LeeWJ. Impact of paraquat regulation on suicide in South Korea. Int J Epidemiol. 2016;45(2):470-479. doi:10.1093/ije/dyv304 |
| 8 | Cha ES, Chang SS, Choi Y, LeeWJ. Trends in pesticide suicide in South Korea, 1983-2014. Epidemiol Psychiatr Sci. 2019;29:e25. doi:10.1017/S2045796019000118 |
| 9 | Kim J, Shin SD, Jeong S, Suh GJ, Kwak YH. Effect of prohibiting the use of Paraquat on pesticide-associated mortality. BMC Public Health. 2017;17(1):858. doi:10.1186/s12889-017-4832-4 |
| 10 | Han DG, Kang SG, Cho SJ, Cho SE, Na KS. Suicide methods according to age and sex: an analysis of data of 239,565 suicide victims in the Republic of Korea from 1991 to 2015. J Nerv Ment Dis. 2018;206(10):770-775. doi: 10.1097/NMD.0000000000000889 |
| 11 | Myung W, Lee GH,Won HH, et al. Paraquat prohibition and change in the suicide rate and methods in South Korea. PLoS One. 2015;10(6):e0128980. doi:10.1371/journal.pone.0128980 |
| 12 | Lee JW, Hwang IW, Kim JW, et al. Common pesticides used in suicide attempts following the 2012 paraquat ban in Korea. J Korean Med Sci. 2015;30(10):1517-1521. doi:10.3346/jkms.2015.30.10.1517 |
| 13 | Lin JJ, Lu TH. Trends in solids/liquids poisoning suicide rates in Taiwan: a test of the substitution hypothesis. BMC Public Health. 2011;11:712. doi:10.1186/1471-2458-11-712 |
| 14 | Chang SS, Lu TH, Eddleston M, et al. Factors associated with the decline in suicide by pesticide poisoning in Taiwan: a time trend analysis, 1987-2010. Clin Toxicol (Phila). 2012;50(6):471-480.  doi:10.3109/15563650.2012.688835 |
| 15 | Chen YY, Kwok CL, Yip PS,Wu KC. A test of the substitution hypothesis: an analysis of urban and rural trends in solid/liquid poisoning suicides in Taiwan. Soc Sci Med. 2013;96:45-51. doi:10.1016/j.socscimed.2013.06.031 |
| 16 | Berecz R, C.ceresM, Szlivka A, et al. Reduced completed suicide rate in Hungary from 1990 to 2001: relation to suicide methods. J Affect Disord. 2005;88(2):235-238. doi:10.1016/j.jad.2005.07.005 |
| 17 | Qin P, Du M,Wang S, et al. The waterfall pattern of suicide mortality in Inner Mongolia for 2008-2015. J Affect Disord. 2019;256:331-336. doi:10.1016/j.jad.2019.05.057 |
| 18 | Gunnell D, Fernando R, Hewagama M, PriyangikaWD, Konradsen F, Eddleston M. The impact of pesticide regulations on suicide in Sri Lanka. Int J Epidemiol. 2007;36(6):1235-1242. doi:10.1093/ije/dym164 |
| 19 | de Silva VA, Senanayake SM, Dias P, Hanwella R. From pesticides to medicinal drugs: time series analyses of methods of self-harm in Sri Lanka. BullWorld Health Organ. 2012;90(1):40-46. doi:10.2471/BLT.11.091785 |
| 20 | Knipe DW,Metcalfe C, Fernando R, et al. Suicide in Sri Lanka 1975-2012: age, period and cohort analysis of police and hospital data. BMC Public Health. 2014;14:839. doi:10.1186/1471-2458-14-839 |
| 21 | Knipe DW, Chang SS, Dawson A, et al. Suicide prevention through means restriction: impact of the 2008-2011 pesticide restrictions on suicide in Sri Lanka. PLoS One. 2017;12(3):e0172893. doi:10.1371/journal.pone.0172893 |
| 22 | Arya V, Page A, Gunnell D, et al. Suicide by hanging is a priority for suicide prevention: method specific suicide in India (2001-2014). J Affect Disord. 2019;257:1-9. doi:10.1016/j.jad.2019.07.005 |
| 23 | Chowdhury FR, Dewan G, Verma VR, et al. Bans of WHO class I pesticides in Bangladesh-suicide prevention without hampering agricultural output. Int J Epidemiol. 2018;47(1):175-184. doi:10.1093/ije/dyx157 |
| 24 | Abu al-Ragheb SY, Salhab AS. Pesticide mortality. A Jordanian experience. Am J Forensic Med Pathol 1989; 10: 221–25. |
| 25 | Knipe DW, Gunnell D, Eddleston M. Preventing deaths from pesticide self-poisoning-learning from Sri Lanka’s success. Lancet Glob Health 2017; 5: e651–52. |
| 26 | Chaparro-Narvaez P, Castaneda-Orjuela C. Mortality due to pesticide poisoning in Colombia, 1998-2011. Biomedica 2015; 35: 90–102. |
| 27 | Berecz R, Szlivka A, Degrell I. [Characteristic features of completed suicides in Hungary between 1994–2001]. A befejezett ongyilkossagok jellemzoi Magyarorszagon 2003; 18: 399–407. |
| 28 | Nandi DN, Mukherjee SP, Banerjee G, Boral GC, Chowdhury A, Bose J. Is suicide preventable by restricting the availability of lethal agents? A rural survey of West Bengal. Indian J Psychiatry 1979; 21: 251–55. |
| 29 | Frost J, Poulsen E. Poisoning due to parathion and other organophosphorus insecticides in Denmark. Dan Med Bull 1964; 11: 169–77. |
| 30 | Casey P, Vale JA. Deaths from pesticide poisoning in England and Wales: 1945-1989. Hum Exp Toxicol 1994; 13: 95–101. |
| 31 | Ito T, Nakamura Y. Deaths from pesticide poisoning in Japan, 1968–2005: data from vital statistics. J Rural Med 2008; 3: 5–9. |
| 32 | Nagami H, Maejima F, Nishigaki Y, Natsukawa S. Trends in paraquat poisoning in Japan—viewed from surveys on clinical cases. J Rural Med 2013; 8: 228–32. |
| 33 | Copeland AR. Organophosphate related fatalities—a violitional biohazard? Forensic Sci Int 1988; 39: 155–62. |
| 34 | Langley R, Sumner D. Pesticide mortality in the United States 1979-1998. Vet Hum Toxicol 2002; 44: 101–05. |
| 35 | Sudakin DL, Power LE. Organophosphate exposures in the United States: a longitudinal analysis of incidents reported to poison centers. J Toxicol Environ Health A 2007; 70: 141–47. |
| 36 | Vijayakumar L, Babu RS. Does ‘no pesticide’ reduce suicides? Int J Soc Psychiatr 2009; 55: 401–06. |
| 37 | Vijayakumar, L., Jeyaseelan, L., Kumar, S., Mohanraj, R., Devika, S., & Manikandan, S.  (2013). A central storage facility to reduce pesticide suicides—A feasibility study from India. BMC Public Health, 13, |
| 38 | Eddleston, M., Adhikari, S., Egodage, S., Ranganath, H., Mohamed, F., Manuweera, G., et al. (2012). Effects of a provincial ban of two toxic organophosphorus insecticides on pesticide poisoning hospital admissions. Clinical Toxicology, 50, 202–209.  <https://doi.org/10.3109/15563650>. 2012.660573 |
| 39 | Gunnell, D., Knipe, D., Chang, S.-S., Pearson, M., Konradsen, F., Lee, W. J., et al.  (2017). Prevention of suicide with regulations aimed at restricting access to highly hazardous  pesticides: A systematic review of the international evidence. Lancet Global Health, 5, e1026–e1037. https://doi.org/10.1016/S2214-109X(17)30299-1 |
| 40 | Hawton, K., Ratnayeke, L., Simkin, S., Harriss, L., & Scott, V. (2009). Evaluation of  acceptability and use of lockable storage devices for pesticides in Sri Lanka that might assist in prevention of self-poisoning. BMC Public Health, 9, 69. https://doi.org/10.1186/1471-2458-9-69 |
| 41 | Bowles J. Suicide in Western Samoa: an example of a suicide prevention program in a developing country. In: Diekstra R, Gulbinat R, De Leo D, Kienhorst I, eds. Preventive Strategies on Suicide. Leiden, the Netherlands: Brill; 1995. |
| 42 | Yan Y, Jiang Y, Liu R, Eddleston M, Tao C, Page A, et al. Impact of pesticide regulations on mor­tality from suicide by pesticide in China: an interrupted time series analysis. Front Psychiatry. 2023;14:1189923 https://doi.org/10.3389/fpsyt.2023.1189923 |
| 43 | Arya V, Page A, Gunnell D, Armstrong G. Changes in method specific suicide following a national pesticide ban in India (2011–2014). J Affect Disord. 2021;278:592–600. https://doi.org/10.1016/j. jad.2020.09.085 |
| 44 | Bonvoisin T, Utyasheva L, Knipe D, Gunnell D, Eddleston M. Suicide by pesticide poisoning in India: a review of pesticide regulations and their impact on suicide trends. BMC Public Health. 2020;20(1):251. https://doi.org/10.1186/s12889-020-8339-z |
| 45 | Eddleston M, Nagami H, Lin CY, Davis ML, Chang SS. Pesticide use, agricultural outputs, and pesticide poisoning deaths in Japan. Clin Toxicol (Phila). 2022;60(8):933–41. |
| 46 | Chang SS, Lin C-Y, Lee MB, Shen LJ, Gunnell D, Eddleston M. The early impact of Paraquat ban on suicide in Taiwan. Clin Toxicol (Phila). 2022;60(1):131–5. https://doi.org/10.1080/15563650.2021.1937 642 |
| 47 | Lin CY, Hsu CY, Lee M-B, Chang SS. Impact of the Paraquat ban on reducing suicide in Taiwan: the effect on 2020 suicide rates. J Suicidology. 2022;17(1):80–7. |

| **Figure S4 node label** | **Corresponding reference** |
| --- | --- |
| 1 | Kreitman N. The coal gas story: United Kingdom suicide rates, 1960-71. Br J Prev Soc Med. 1976;30(2):86-93. doi:10.1136/jech.30.2.86 |
| 2 | Kreitman N, Platt S. Suicide, unemployment, and domestic gas detoxification in Britain. J Epidemiol Community Health. 1984;38(1):1-6. doi:10.1136/jech.38.1.1 |
| 3 | Gunnell D, Middleton N, Frankel S. Method availability and the prevention of suicide—a re-analysis of secular trends in England andWales 1950-1975. Soc Psychiatry Psychiatr Epidemiol. 2000;35(10):437-443. doi:10.1007/s001270050261 |
| 4 | Clarke RV, Mayhew PAT. Crime as opportunity: a note on domestic gas suicide in Britain and the Netherlands. Br J Criminol. 1989;29(1):35-46. doi:10.1093/oxfordjournals.bjc.a047788 |
| 5 | Thomas K, Gunnell D. Suicide in England andWales 1861-2007: a time-trends analysis. Int J Epidemiol. 2010; 39(6):1464-1475. doi:10.1093/ije/dyq094 |
| 6 | Hassall C, Trethowan WH. Suicide in Birmingham. BMJ. 1972;1(5802):717-718. doi:10.1136/bmj.1.5802.717 |
| 7 | Lester D, Hodgson J. The effects of the detoxification of domestic gas on the suicide rate in Scotland. Eur J Psychiatry. 1992;6(3):171-174. |
| 8 | Curran PS, Lester D. Trends in the methods used for suicide in Northern Ireland. Ulster Med J. 1991;60(1): 58-62. |
| 9 | Dervic K, Friedrich E, Prosquill D, et al. Suicide among Viennese minors, 1946-2002. Wien KlinWochenschr. 2006;118(5-6):152-159. doi:10.1007/s00508-006-0567-4 |
| 10 | Wiedenmann A,Weyerer S. The impact of availability, attraction and lethality of suicide methods on suicide rates in Germany. Acta Psychiatr Scand. 1993;88(5):364-368. doi:10.1111/j.1600-0447.1993.tb03474.x |
| 11 | Moens GFG, Loysch MJM, Honggokoesoemo S, van de Voorde H. Recent trends in methods of suicide. Acta Psychiatr Scand. 1989;79(3):207-215. doi:10.1111/j.1600-0447.1989.tb10246.x |
| 12 | Lester D. The effect of the detoxification of domestic gas in Switzerland on the suicide rate. Acta Psychiatr Scand. 1990;82(5):383-384. doi:10.1111/j.1600-0447.1990.tb01406.x |
| 13 | Lester D, Abe K. The effect of restricting access to lethal methods for suicide: a study of suicide by domestic gas in Japan. Acta Psychiatr Scand. 1989;80(2):180-182. doi:10.1111/j.1600-0447.1989.tb01324.x |
| 14 | Lester D. The effects of detoxification of domestic gas on suicide in the United States. Am J Public Health. 1990;80(1):80-81. doi:10.2105/AJPH.80.1.80 |
| 15 | Burvill PW. The changing pattern of suicide by gassing in Australia, 1910-1987: the role of natural gas and motor vehicles. Acta Psychiatr Scand. 1990;81(2):178-184. doi:10.1111/j.1600-0447.1990.tb06475.x |
| 16 | Thomas K, Gunnell D. Suicide in England andWales 1861-2007: a time-trends analysis. Int J Epidemiol. 2010; 39(6):1464-1475. doi:10.1093/ije/dyq094 |
| 17 | Amos T, Appleby L, Kiernan K. Changes in rates of suicide by car exhaust asphyxiation in England andWales. Psychol Med. 2001;31(5):935-939. doi:10.1017/S0033291701003920 |
| 18 | Kendell RE. Catalytic converters and prevention of suicides. Lancet. 1998;352(9139):1525.  doi:10.1016/S0140-6736(05)60332-7 |
| 19 | Skilling GD, Sclare PD,Watt SJ, Fielding S. The effect of catalytic converter legislation on suicide rates in Grampian and Scotland 1980-2003. Scott Med J. 2008;53(4):3-6. doi:10.1258/RSMSMJ.53.4.3 |
| 20 | Hepp U, RingM, Frei A, RösslerW, Schnyder U, Ajdacic-Gross V. Suicide trends diverge by method: Swiss suicide rates 1969-2005. Eur Psychiatry. 2010;25(3):129-135. doi:10.1016/j.eurpsy.2009.05.005 |
| 21 | Lester S, Abe K. Car availability, exhaust toxicity, and suicide. Ann Clin Psychiatry. 1989;1(4):247-250. doi:10.3109/10401238909149990 |
| 22 | Lester D. Changing rates of suicide by car exhaust in men and women in the United States after car exhaust was detoxified. Crisis. 1989;10(2):164-168. |
| 23 | Mott JA,Wolfe MI, Alverson CJ, et al. National vehicle emissions policies and practices and declining US carbon monoxide-related mortality. JAMA. 2002;288(8):988-995. doi:10.1001/jama.288.8.988 |
| 24 | Hampson NB, Holm JR. Suicidal carbon monoxide poisoning has decreased with controls on automobile emissions. Undersea Hyperb Med. 2015;42(2):159-164. |
| 25 | Routley VH, Ozanne-Smith J. The impact of catalytic converters on motor vehicle exhaust gas suicides. Med J Aust. 1998;168(2):65-67. doi:10.5694/j.1326-5377.1998.tb126713.x |
| 26 | McClure GM. Changes in suicide in England and Wales, 1960-1997. Br J Psychiatry. 2000;176:64-67. |
| 27 | Kelly S, Bunting J. Trends in suicide in England and Wales, 1982-96. Popul Trends. Summer 1998:29-41. |
| 28 | Shelef M. Unanticipated benefits of automotive emission control: reduction in fatalities by motor vehicle exhaust gas. Sci Total Environ. 1994;146-147: 93-101. |
| 29 | Sarchiapone M,Mandelli L, IosueM, et al: Controlling access to suicide means. Int J Environ Res Public Health 2011; 8:4550–4562 |
| 30 | Thomsen AH, Gregersen M: Suicide by carbon monoxide from car exhaust gas in Denmark 1995–1999. Forensic Sci Int 2006; 161:41–46 |
| 31 | Routley V: Motor vehicle exhaust gas suicide: review of countermeasures. Crisis 2007; 28(suppl 1):28–35 |
| 32 | Lester D. Effects of detoxification of domestic gas on suicide in the Netherlands. Psychol Rep. 1991; 68:202. |
| 33 | Studdert DM, Gurrin LC, Jatkar U, Pirkis J. Relationship between vehicle emissions laws and incidence of suicide by motor vehicle exhaust gas in Australia, 2001-06: An ecological analysis. PLoS Med 2010;7(1):e1000210. |
| 34 | Nordentoft M, Qin P, Helweg-Larsen K, Juel K. Time-trends in method-specific suicide rates compared with the availability of specific compounds. The Danish experience. Nord J Psychiatry. 2006;60(2):97–106. doi:10.1080/08039480600600169 |
| 35 | Yip, P. S. F., Law, C. K., Fu, K.-W., Law, Y. W., Wong, P. W. C., & Xu, Y. (2010). Restricting the means of suicide by charcoal burning. The British Journal of Psychiatry, |
| 36 | Chen, Y.-Y., Chen, F., Chang, S.-S., Wong, J., & Yip, P. S. F. (2015). Assessing the efficacy of restricting access to barbecue charcoal for suicide prevention in Taiwan: A community-based  intervention trial. PLoS ONE, 10(8), Article e0133809. <https://doi>. org/10.1371/journal.pone.0133809 |
| 37 | Jo, S.-J., Yun, M. K., & Lee, M.-S. (2019). Effects of a province-based strategy to prevent suicide using charcoal burning: A preliminary time series analysis. Psychiatry Investigation, 16(8), 621–624. https://doi.org/10.30773/pi.2019.06.13 |

| **Figure S6 node label** | **Corresponding reference** |
| --- | --- |
| 1 | Hawton K, Simkin S, Deeks J, et al. UK legislation on analgesic packs: before and after study of long term effect on poisonings. BMJ. 2004;329(7474):1076-1079. doi:10.1136/bmj.38253.572581.7C |
| 2 | Hawton K, Bergen H, Simkin S, et al. Long term effect of reduced pack sizes of paracetamol on poisoning deaths and liver transplant activity in England andWales: interrupted time series analyses. BMJ. 2013;346:f403. doi:10.1136/bmj.f403 |
| 3 | Morgan OW, Griffiths C, Majeed A. Interrupted time-series analysis of regulations to reduce paracetamol (acetaminophen) poisoning. PLoS Med. 2007;4(4):e105. doi:10.1371/journal.pmed.0040105 |
| 4 | Hawton K, Bergen H, Simkin S, et al. Effect of withdrawal of co-proxamol on prescribing and deaths from drug poisoning in England andWales: time series analysis. BMJ. 2009;338:b2270. doi:10.1136/bmj.b2270 |
| 5 | Hawton K, Bergen H, Simkin S,Wells C, Kapur N, Gunnell D. Six-year follow-up of impact of co-proxamol withdrawal in England andWales on prescribing and deaths: time-series study. PLoS Med. 2012;9(5):e1001213. doi:10.1371/journal.pmed.1001213 |
| 6 | Sandilands EA, Bateman DN. Co-proxamol withdrawal has reduced suicide from drugs in Scotland. Br J Clin Pharmacol. 2008;66(2):290-293. doi:10.1111/j.1365-2125.2008.03206.x |
| 7 | Delcher C, Chen G,Wang Y, Slavova S, Goldberger BA. Fatal poisonings involving propoxyphene before and after voluntary withdrawal from the United States’ market: an analysis from the state of Florida. Forensic Sci Int. 2017;280:228-232. doi:10.1016/j.forsciint.2017.10.008 |
| 8 | Carlsten A, Allebeck P, Brandt L. Are suicide rates in Sweden associated with changes in the prescribing of medicines? Acta Psychiatr Scand. 1996;94(2):94-100. doi:10.1111/j.1600-0447.1996.tb09831.x |
| 9 | Lester D, Abe K. The effect of controls on sedatives and hypnotics on their use for suicide. J Toxicol Clin Toxicol. 1989;27(4-5):299-303. doi:10.3109/15563658908994427 |
| 10 | Oliver RG, Hetzel BS. Rise and fall of suicide rates in Australia: relation to sedative availability. Med J Aust. 1972;2(17):919-923. doi:10.5694/j.1326-5377.1972.tb103635.x |
| 11 | Thelander G, Jönsson AK, Personne M, Forsberg GS, Lundqvist KM, Ahlner J. Caffeine fatalities—do sales restrictions prevent intentional intoxications? Clin Toxicol (Phila). 2010;48(4):354-358. doi:10.3109/15563650903586752 |
| 12 | Prince M, Thomas S, James O, Hudson M. Reduction in incidence of severe paracetamol poisoning. Lancet 2000; 355: 2047–2048. |
| 13 | Turvill J, Burroughs A, Moore K. Change in occurrence of paracetamol overdose in UK after introduction of blister packs. Lancet 2000; 355: 2048–2047. |
| 14 | Robinson D, Smith A, Johnston, G. D. Severity of overdose after restriction of paracetamol availability: retrospective study. Br Med J 2000; 321: 926–927. |
| 15 | Hawton K, Townsend E, Deeks J et al. Effects of legislation restricting pack sizes of paracetamol and salicylate on self-poisoning in the United Kingdom: before and after study.  Br Med J 2001; 322: 1–7. |
| 16 | Sheen C, Dillon J. The effect on toxicity and healthcare costs on reducing the size of available acetaminophen pack sizes in the Tayside region of Scotland. Gastroenterology 2001; 120(Suppl. 1):A-228. |
| 17 | Sheen C, Dillon J, Bateman N, Simpson K, Macdonald T. Paracetamol pack size restriction: the impact on paracetamol poisoning and the over-the-counter supply of paracetamol, aspirin and ibuprofen. Pharmacoepidemiol Drug Safety 2002; 11: 329–331. |
| 18 | Bateman N, Bain M, Gorman D, Murphy D. Changes in paracetamol, antidepressant and opioid poisoning in Scotland during the 1990s. Q J Med 2003; 96: 125–132. |
| 19 | Sheen C, Dillon J, Bateman N, Simpson K, MacDonald T. Paracetamol-related deaths in Scotland, 1994–2000. Br J Clin Pharmacol 2002; 54: 430–432. |
| 20 | Hughes B, Durran A, Langford N, Mutimer D. Paracetamol poisoning – impact of pack size restrictions. J Clin Pharm Therap 2003; 28: 307–310. |
| 21 | Donohoe E, Tracey J. Restrictions on sale of paracetamol in Ireland had no impact on the number of tablets ingested in acute deliberate overdose. J Toxicol Clin Toxicol 2001; 38: 251. |
| 22 | Laing W, Gordon L, Lee D, Good A, Bateman D. Have the new pack size regulations impacted on UK paracetamol overdose? J Toxicol Clin Toxicol 2001; 39: 301. |
| 23 | Thomas M, Jowett N. Severity of overdose after restriction of paracetamol availability. Restriction has not reduced admissions with self-poisoning. Br Med J 2001; 322: 554. |
| 24 | Hawton K, Bergen H, Simkin S, et al. Impact of diff erent pack sizes of paracetamol in the United Kingdom and Ireland on intentional overdoses: a comparative study. BMC Public Health 2011; 11: 460. |
| 25 | Nordentoft M, Qin P, Helweg-Larsen K, Juel K. Restrictions in means for suicide: an eff ective tool in preventing suicide: the Danish experience. Suicide Life Threat Behav 2007; 37: 688–97. |
| 26 | Crome P. The toxicity of drugs used for suicide. Acta Psychiatr Scand Suppl. 1993;371:33-37. |
| 27 | Nielsen AS, Nielsen B. Pattern of choice in preparation of attempted suicide by poisoning with particular reference to changes in the pattern of prescriptions [in Dutch]. Ugeskr Laeger. 1992;154:1972-1976. |
| 28 | Yamasawa K, Nishimukai H, Ohbora Y, Inoue K. A statistical study of suicides through intoxication. Acta Med Leg Soc (Liege). 1980;30:187-192. |
| 29 | Hawton K. United Kingdom legislation on pack sizes of analgesics: background, rationale, and effects on suicide and deliberate self-harm. Suicide Life Threat Behav. 2002;32:223-229. |
| 30 | Whitlock FA. Suicide in Brisbane, 1956 to 1973: the drug-death epidemic.MedJAust. 1975;1:737-743. |
| 31 | Retterstol N. Norwegian data on death due to overdose of antidepressants. Acta Psychiatr Scand. 1989;80(suppl 354):61-68. |
| 32 | Wheeler BW, Metcalfe C, MartinRM, Gunnell D. International impacts of regulatory action to limit antidepressant prescribing on rates |
| 33 | Wheeler BW, Gunnell D, Metcalfe C, Stephens P, Martin RM. The population impact on incidence of suicide and non-fatal self harm of regulatory action against the use of selective serotonin reuptake inhibitors in under 18s in the United Kingdom: ecological study. BMJ 2008;336(7643):542–5. |

| **Figure S8 node label** | **Corresponding reference** |
| --- | --- |
| 1 | Hahn RA, Bilukha O, Crosby A, et al. Firearms laws and the reduction of violence: a systematic review. Am J Prev Med 2005; 28: 40–71. |
| 2 | Rodriguez Andres A, Hempstead K. Gun control and suicide: the impact of state fi rearm regulations in the United States, 1995–2004. Health Policy 2011; 101: 95–103. |
| 3 | Klieve H, Barnes M, De Leo D. Controlling fi rearms use in Australia: has the 1996 gun law reform produced the decrease in rates of suicide with this method? Soc Psychiatry Psychiatr Epidemiol 2009; 44: 285–92. |
| 4 | Rosengart M, Cummings P, Nathens A, Heagerty P, Maier R, Rivara F. An evaluation of state firearm regulations and homicide and suicide death rates. Inj Prev 2005; 11: 77–83. |
| 5 | Fleegler EW, Lee LK, Monuteaux MC, Hemenway D, Mannix R. Firearm legislation and firearm-related fatalities in the United States. JAMA Intern Med 2013; 173: 732–40. |
| 6 | Gjertsen F, Leenaars A, Vollrath ME. Mixed impact of firearms restrictions on fatal fi rearm injuries in males: a national observational study. Int J Environ Res Public Health 2014; 11: 487–506. |
| 7 | Reisch T, Steff en T, Habenstein A, Tschacher W. Change in suicide rates in Switzerland before and after fi rearm restriction resulting from the 2003 “Army XXI” reform. Am J Psychiatry 2013; 170: 977–84. |
| 8 | Lubin G, Werbeloff N, Halperin D, Shmushkevitch M, Weiser M, Knobler HY. Decrease in suicide rates after a change of policy reducing access to fi rearms in adolescents: a naturalistic epidemiological study. Suicide Life Threat Behav 2010; 40: 421–24. |
| 9 | Beautrais AL, Fergusson DM, Horwood LJ. Firearms legislation and reductions in firearm-related suicide deaths in New Zealand. Aust N Z J Psychiatry 2006; 40: 253–59. |
| 10 | Kapusta ND, Etzersdorfer E, Krall C, Sonneck G. Firearm legislation reform in the European Union: impact on fi rearm availability, firearm suicide and homicide rates in Austria. Br J Psychiatry 2007; 191: 253–57. 27 McPhedran S, |
| 11 | McPhedran S, Baker J. Suicide prevention and method restriction: evaluating the impact of limiting access to lethal means among young Australians. Arch Suicide Res 2012; 16: 135–46. |
| 12 | Grossman DC, Mueller BA, Riedy C, et al. Gun storage practices and risk of youth suicide and unintentional fi rearm injuries. JAMA 2005; 293: 707–14. |
| 13 | Loftin C, McDowall D, Wiersema B, Cottey TJ. Effects of restrictive licensing of handguns on homicide and suicide in the District of Columbia. N Engl J Med. 1991;325:1615-1620. |
| 14 | Bridges FS, Kunselman JC. Gun availability and use of guns for suicide, homicide, and murder in Canada. Percept Mot Skills. 2004;98:594-598. |
| 15 | Lester D, Leenaars A. Suicide rates in Canada before and after tightening firearm control laws. Psychol Rep. 1993;72:787-790. |
| 16 | Snowdon J, Harris L. Firearms suicides in Australia. Med J Aust. 1992;156:79-83. |
| 17 | Ludwig J, Cook PJ. Homicide and suicide rates associated with implementation of the Brady Handgun Violence Prevention Act. JAMA. 2000;284:585-591. |
| 18 | Carrington PJ. Gender, gun control, suicide and homicide in Canada. Arch Suicide Res. 1999;5:71-75. 79. Kreitman N. The coal gas story: United Kingdom suicide rates, 1960-71. Br J Prev Soc Med. 1976;30: 86-93. |
| 19 | Cantor CH, Slater PJ. The impact of firearm control legislation on suicide in Queensland: preliminary findings. Med J Aust. 1995;162:583-585. |
| 20 | Mann JJ, Michel CA: Prevention of firearm suicide in the United States: what works and what is possible. Am J Psychiatry 2016; 173:969–979 |
| 21 | McGinty EE, Webster DW, Barry CL: Gun policy and serious mental illness: priorities for future research and policy. Psychiatr Serv 2014; 65:50–58 |
| 22 | Caron J. Gun control and suicide: possible impact of Canadian legislation to ensure safe storage of firearms. Arch Suicide Res 2004;8(4):361–74. |
| 23 | Cheung AH, Dewa CS. Current trends in youth suicide and firearms regulations. Can J Public Health 2005;96(2):131–5. |
| 24 | Leenaars AA, Lester D. The impact of gun control on suicide and homicide across the life span. Can J Behav Sci 1997;29(1):1–6. |
| 25 | Niederkrotenthaler T, Till B, Herberth A, et al. Can media effects counteract legislation reforms? The case of adolescent firearm suicides in the wake of the Austrian firearm legislation. J Adolesc Health 2009;44(1):90–3. |
| 26 | Kleck G, Patterson EB. The impact of gun control and gun ownership levels on violence rates. J Quantitative Criminol 1993;9:249–87. |
| 27 | McDowall D, Loftin C, Wiersema B. Using quasi-experiments to evaluate firearm laws: comment on Britt et al.’s reassessment of the D.C. gun law. Law Society Rev 1996;30:381–91. |
| 28 | Lott JR, Whitley JE. Safe-storage gun laws: accidental deaths, suicides, and crime. J Law Econ 2001;44:659 –90. |
| 29 | Magaddino JP, Medoff MH. An empirical analysis of federal and state firearm control laws. In: Kates DB, ed. Firearms and violence. Cambridge MA: Ballinger, 1984:225–58. |
| 30 | DeZee MR. Gun control legislation: impact and ideology. Law Policy Q 1983;5:367–79. |
| 31 | Murray D. Handguns, gun control laws and firearm violence. Social Problems 1975;23:81–92. |
| 32 | Cummings P, Grossman DC, Rivara FP, Koepsell TD. State gun safe storage laws and child mortality due to firearms. JAMA 1997;278:1084–6. |
| 33 | Canadian Department of Justice. A statistical analysis of the impacts of the 1977 firearms control legislation. Ottawa: Department of Justice, Programme Evaluation Section, 1996. |
| 34 | Boor M, Blair JH. Suicide rates, handgun control laws, and sociodemographic variables. Psychol Rep 1990;66:923–30. |
| 35 | Geisel M, Roll R, Wettick R. The effectiveness of state and local regulation of handguns. Duke Law J 1969;43:647–73. |
| 36 | Medoff MH, Magaddino JP. Suicides and firearm control laws. Evaluation Rev 1983;7:357–72. |
| 37 | Sloan JH, Rivara FP, Reay DT, Ferris JAJ, Path MRC, Kellermann AL. Firearm regulations and rates of suicide: a comparison of two metropolitan areas. N Engl J Med 1990;322:369 –73. |
| 38 | Anestis MD, Selby EA, and Butterworth SE (2017) Rising longitudinal trajectoires in suicide rates: The role of firearm suicide rates and firearm legislation. Preventive medicine 100, 159-166 |

References

1. Spittal M, Pirkis J, Gurrin L. Meta-analysis of incidence rate data in the presence of zero events. BMC MEDICAL RESEARCH METHODOLOGY. 2015;15. doi: 10.1186/s12874-015-0031-0. PubMed PMID: WOS:000353939800001.
